# Muscleblind-like proteins use modular domains to localize RNAs by riding kinesins and docking to membranes

**DOI:** 10.1038/s41467-023-38923-6

**Published:** 2023-06-09

**Authors:** Ryan P. Hildebrandt, Kathryn R. Moss, Aleksandra Janusz-Kaminska, Luke A. Knudson, Lance T. Denes, Tanvi Saxena, Devi Prasad Boggupalli, Zhuangyue Li, Kun Lin, Gary J. Bassell, Eric T. Wang

**Affiliations:** 1 Department of Molecular Genetics & Microbiology, Center for Neurogenetics, Genetics Institute, University of Florida, Gainesville, FL USA; 2 Department of Cell Biology, Emory University School of Medicine, Atlanta, GA USA; 3 Myology Institute, University of Florida, Gainesville, FL USA

**Keywords:** Cellular imaging, RNA transport, Kinesin

## Abstract

RNA binding proteins (RBPs) act as critical facilitators of spatially regulated gene expression. Muscleblind-like (MBNL) proteins, implicated in myotonic dystrophy and cancer, localize RNAs to myoblast membranes and neurites through unknown mechanisms. We find that MBNL forms motile and anchored granules in neurons and myoblasts, and selectively associates with kinesins Kif1bα and Kif1c through its zinc finger (ZnF) domains. Other RBPs with similar ZnFs associate with these kinesins, implicating a motor-RBP specificity code. MBNL and kinesin perturbation leads to widespread mRNA mis-localization, including depletion of *Nucleolin* transcripts from neurites. Live cell imaging and fractionation reveal that the unstructured carboxy-terminal tail of MBNL1 allows for anchoring at membranes. An approach, termed RBP Module Recruitment and Imaging (RBP-MRI), reconstitutes kinesin- and membrane-recruitment functions using MBNL-MS2 coat protein fusions. Our findings decouple kinesin association, RNA binding, and membrane anchoring functions of MBNL while establishing general strategies for studying multi-functional, modular domains of RBPs.

## Abstract (web summary)

RNA localization is mediated by kinesin motors and anchoring. However, mechanisms underlying specificity are unclear. Here, the authors find that MBNL protein’s zinc fingers prefer specific kinesins, and its unstructured tail mediates membrane anchoring.

## Introduction

Cells contain highly organized microenvironments in which mRNA localization and translation are spatiotemporally regulated to influence cell structure and function^1^. This regulation is essential in highly polarized and differentiated cell types such as multinucleated skeletal muscle and neurons^2^. mRNA *cis*-elements, often in 3′ UTRs, recruit *trans*-acting RNA binding proteins (RBPs), facilitating assembly of ribonucleoprotein (RNP) granules competent for transport and local translation^3^. Transport of RNPs often depends on kinesins travelling along microtubules. Indeed, over 40 RBPs are associated with conventional kinesin-1 (KIF5)^4^. For example, ZBP1 (IGF2BP1) associates with KIF5 and KIF11 to regulate β-actin mRNA localization and local translation in neurons and fibroblasts, respectively^5–8^, and FMRP associates with the kinesin light chain of KIF5 to transport RNA and regulate local translation in an activity-dependent manner^9,10^. Recently, RNAs and translational machinery were shown to travel with late endosomes and lysosomes via adapters such as annexin A11^11,12^, implicating membrane-bound vesicular trafficking as yet another localization mechanism. Despite critical roles for all these components to regulate mRNA transport, it remains unclear whether a code exists to specify RBP-motor associations. An open question is whether certain kinesin tails can associate with certain RBP domains, facilitating specificity for transport, anchoring, and response to physiological cues. Because many RBPs recognize specific mRNAs via sequence and/or structure, kinesin association and transport behavior may be influenced by RNP composition.

Genetic disruptions to kinesins and RBPs can cause disease. Loss of the RNA binding protein, FMRP, causes fragile X syndrome (FXS), concomitant with impairment of mRNA localization and local translation^13^. Mutations in kinesins are linked to Charcot-Marie-Tooth disease^14^, hereditary spastic paraplegia^15,16^, and amyotrophic lateral sclerosis^17,18^. In the repeat expansion disease myotonic dystrophy (*dystrophia myotonica*, DM), expanded CTG or CCTG repeats are transcribed into toxic RNA that form intranuclear foci, sequestering and functionally depleting the Muscleblind-like (MBNL) family of RBPs^19^. MBNLs are deeply conserved throughout metazoa, and mammals encode 3 paralogs: MBNL1, MBNL2, and MBNL3^20–22^. MBNL1 shows highest expression in heart and muscle, MBNL2 in brain^21,23^, and MBNL3 in placenta and muscle satellite cells^24^. In the nucleus, MBNLs regulate alternative splicing^25,26^ and polyadenylation^27^, and loss of MBNL activity in DM causes mis-splicing and disease symptoms, such as muscle weakness, myotonia, and cardiac arrhythmia^28–30^. In the cytoplasm, MBNLs regulate mRNA localization^26,31,32^ by enhancing association of target RNAs to membranes in myoblasts, particularly those encoding secreted proteins^26^. In neuronal culture, MBNLs localize alternative 3’ UTR isoforms to neurites^32^, while in rat hippocampal neuropil, localized RNAs are enriched for MBNL motifs^33^. Indeed, a transgenic mouse expressing expanded CTG repeats showed reduced levels of cytoplasmic MBNL1 in dendrites^34^, and CTG repeat expression in cultured neurons affected neurite outgrowth^35^. This defect was rescued by co-expression of cytoplasmic but not nuclear MBNL1^35^. Together, these findings suggest that depletion of cytoplasmic MBNLs may also drive disease symptoms.

Despite established roles for MBNLs to localize mRNAs in various cell types, we lack mechanistic insight into whether MBNL-containing RNPs associate with motors and how they may anchor to membranes. MBNLs possess two pairs of zinc fingers (ZnFs) separated by an unstructured linker, as well as an unstructured C-terminal domain. Specificity for mRNA targets is achieved via the ZnFs^26,36,37^, with ZnF1/2 of MBNL1 showing greater specificity for YGCY motifs than ZnF3/4^38^. Both MBNL1 and MBNL2 are extensively alternatively spliced, with 9 alternative isoforms encoded by MBNL1 alone; it is also unknown whether specific isoforms show preferences for motor association or membrane anchoring activities.

Here, we have used molecular, cellular, imaging, and computational approaches to elucidate molecular mechanisms for MBNL-mediated RNA localization. MBNL1 and MBNL2 form granules exhibiting processive motility in neurons, with MBNL1 exhibiting pronounced anchoring behavior in myoblasts. A kinesin recruitment assay^39,40^ revealed that Kif1bα and Kif1c tail domains selectively associate with MBNL zinc fingers in an RNA-binding-independent manner. This association results in co-transport of MBNLs and kinesins in live neurons and their co-immunoprecipitation in cell culture. We also found that Tis11d and Cpsf4, RBPs with zinc fingers structurally similar to MBNL, also associate with these kinesins, suggesting a structure-function relationship among RBP domains and kinesin tails. Dominant negative expression of specific kinesin tails or functional depletion of MBNL by CTG repeat over-expression, followed by subcellular fractionation and RNA-seq, revealed transcriptome-wide mis-localization of mRNAs in neuronal cell lines and depletion of nucleolin mRNA from neurites. Cellular fractionation and live imaging revealed that the unstructured C-terminal tail of MBNL facilitates membrane anchoring and recruitment of an mRNA reporter to membranes, defining a function for this domain and providing mechanistic basis for previous observations. mRNA binding and kinesin association were decoupled using a reconstituted synthetic system (RBP-MRI), highlighting the multi-functional modularity of RBPs, and providing a platform for future studies of other RBP-kinesin pairs. These findings indicate that certain kinesins prefer certain RBP domains and implicate a modular kinesin-RBP code, with broad implications for mRNP trafficking to distal locations in diverse cell types and tissues.

## Results

### MBNL proteins form motile granules in primary neurons and myoblasts

**Fig. 1:**
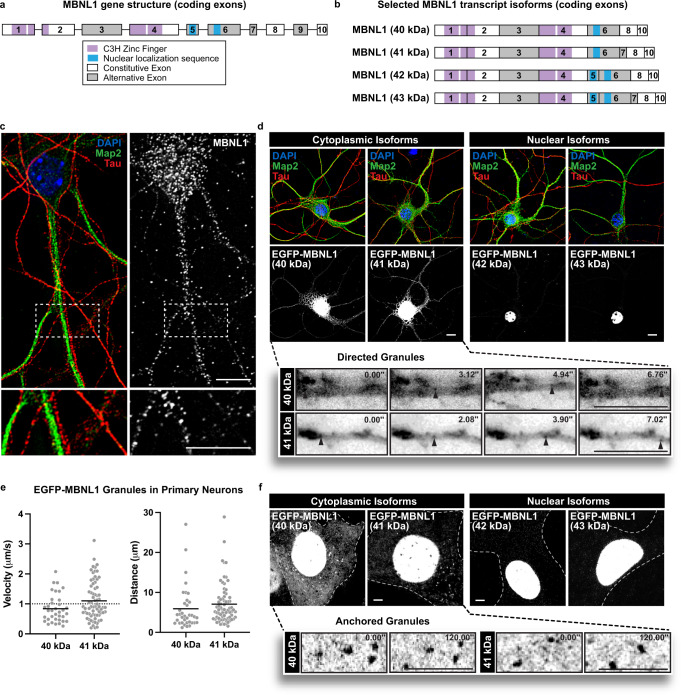
Cytoplasmic MBNL1 granules exhibit directed motion in neurons and anchoring in C2C12 myoblasts. **a** Exon and protein domain structure of MBNL1, and (**b**), isoforms studied. **c** Representative image showing MBNL1 granules (white) in axons (Tau, red) and dendrites (Map2, green) of 9 DIV cultured primary mouse cortical neurons; nuclei (blue) labeled with DAPI. Scale bar = 10 µm. **d** (Above) Representative images of EGFP-MBNL1 40, 41, 42, and 43 kDa isoforms (white) in 8 DIV cultured primary mouse cortical neurons; nuclei (blue) labeled with DAPI, axons and dendrites labeled with Tau (red) and Map2 (green), respectively. Scale bar = 10 µm. (Below) Representative time lapse images of motile EGFP-MBNL1 40 kDa and 41 kDa granules in live mouse cortical neurons. Time (sec) indicated in top right. Scale bars = 10 µm. **e** Quantitation of speeds and distances traveled by cytoplasmic MBNL1 granules (MBNL1–40 kDa − 36 tracks, 1.57 tracks/cell; MBNL1–41 kDa − 71 tracks, 2.37 tracks/cell) in primary mouse cortical neurons. Dotted line in velocity chart represents typical speed of microtubule-dependent transport. Track data come from 3 biological replicates. **f** (Above) Representative images of EGFP-MBNL1 40, 41, 42, and 43 kDa in C2C12 myoblasts. Dotted lines outline cell boundaries. (Below) Representative time lapse images of anchored GFP-MBNL1 40 kDa and 41 kDa granules in C2C12 myoblasts. Time (sec) indicated in top right. Scale bars = 5 µm.

We first assessed the protein expression and distribution of MBNL1 and MBNL2 in mouse cultured primary neurons by immunofluorescence (IF). Both proteins increase in abundance during culture (most dramatically between 8 and 10 days in vitro, DIV), consistent with previous reports of increased mRNA expression^21^. Enrichment of endogenous MBNL2 was observed in the nucleus, whereas MBNL1 showed more cytoplasmic localization (Supplementary Fig. 1a). Similar localization patterns were demonstrated by EGFP-MBNL1 fusions, where isoforms lacking exon 5, which contains a nuclear localization signal (NLS), were more cytoplasmic, and those containing exon 5 were more nuclear (Fig. 1a, b, d). Higher resolution imaging in 10 DIV neurons revealed MBNL1 and MBNL2 puncta in both axons and dendrites, reminiscent of other RBPs known to regulate mRNA localization^41,42^ (Fig. 1c; Supplementary Fig. 1c).

Granules containing EGFP-MBNL1 isoform fusions lacking exon 5 exhibited directed, bi-directional movements in axons and dendrites in 8 DIV neurons (Fig. 1d; Supplementary Movie 1). There was no preference for anterograde vs. retrograde trajectories (data not shown). These motile granules moved with velocities consistent with active transport (MBNL1 40 kDa mean velocity ± SEM: 0.84 ± 0.08 μm/sec, MBNL1–41 kDa mean velocity ± SEM: 1.10 ± 0.08 μm/sec; Fig. 1e), often displaying long, directed runs (MBNL1–40 kDa mean distance ± SEM: 5.92 ± 0.92 μm, MBNL1–41 kDa mean distance ± SEM: 7.06 ± 0.60 μm, Fig. 1e). Again, MBNL2 was predominantly found in the nucleus of primary neurons (Supplementary Fig. 1c), but EGFP-MBNL2 isoform fusions lacking exon 5 could be observed in the cytoplasm and showed similar motility (Mean Velocities ± SEM – MBNL2-isoform 1: 1.21 ± 0.09 μm/sec, MBNL2-isoform 3: 1.00 ± 0.08 μm/sec, MBNL2-isoform 4: 1.16 ± 0.05 μm/sec; Mean Distances ± SEM – MBNL2-isoform 1: 7.62 ± 0.75 μm, MBNL2-isoform 3: 6.36 ± 0.73 μm, MBNL2-isoform 4: 8.30 ± 0.57 μm, Supplementary Fig. 1d, e).

Live cell imaging of EGFP-MBNL1 fusions in C2C12 myoblasts confirmed nuclear vs. cytoplasmic localization patterns for isoforms with or without exon 5, respectively. In these cells, most particles were anchored, with rare instances of fast diffusive or directed motion (Fig. 1f; Supplementary Movie 2). Together, these observations suggested that MBNL-containing granules are actively transported by molecular motors, yet also possess features that facilitate anchoring to stable, intracellular structures. We subsequently explored mechanisms mediating both of these behaviors in myoblast and neuronal systems.

### MBNL1 associates with kinesin-3 family members Kif1bα and Kif1c for transport

**Fig. 2:**
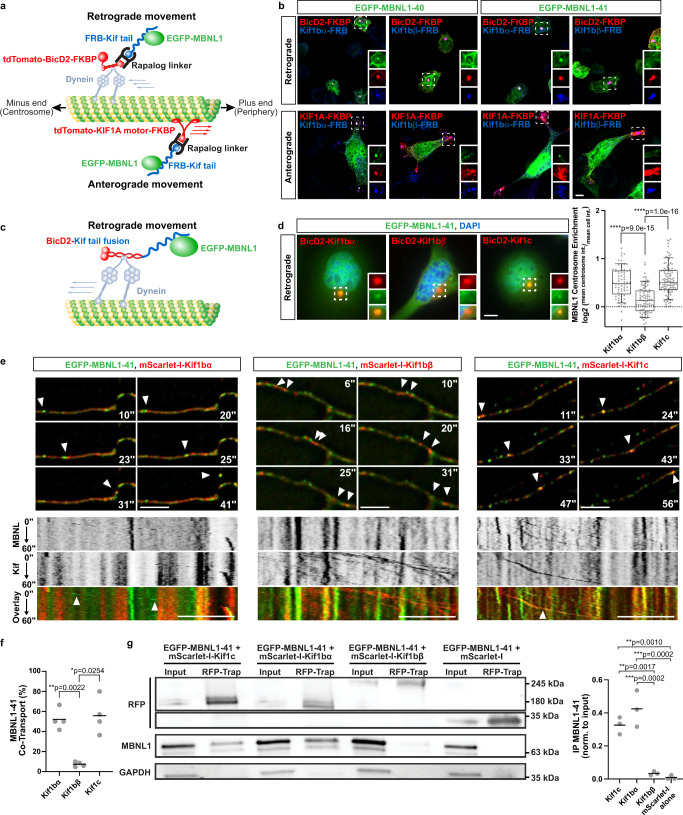
MBNL1 associates, co-transports, and co-immunoprecipitates with Kif1bα and Kif1c. **a** Split kinesin recruitment assay used to identify MBNL1-kinesin associations. Cargoes are recruited in a retrograde or anterograde direction in a rapalog-dependent manner, depending on choice of motor complex. **b** Representative images showing recruitment of EGFP-MBNL1 (green) to the centrosome by Kif1bα, but not Kif1bβ, using Myc-tagged, FRB-kinesin tail fusions (blue) co-expressed with BicD2-FKBP or KIF1A motor-FKBP (red) in N2A cells. Scale bar = 10 µm. **c** A modified kinesin recruitment assay that uses BicD2-kinesin tail fusions to further characterize MBNL1-kinesin associations. **d** Representative images and quantitation in N2A cells of EGFP-MBNL1 (green) at the centrosome when co-expressed with BicD2-kinesin tail fusions (red); nuclei (blue) labeled with DAPI; Kif1bα: *n* = 93 cells (50, 20, and 23 cells in each replicate), Kif1bβ: *n* = 81 cells (25, 16, and 40 cells in each replicate), Kif1c: *n* = 112 cells (53, 19, and 40 cells in each replicate); across 3 biological replicates. Scale bar = 10 µm. Bars represent median, box outlines represent upper and lower quartiles, whiskers represent 10th and 90th percentiles. Dotted line represents no enrichment. ****p < 0.0001 by two-tailed Mann–Whitney *U* test. **e** Representative dual color time lapse images of EGFP-MBNL1 (green) and mScarlet-I-kinesins (red) in live 8 DIV primary mouse cortical neurons. Arrows denote co-transported, anterograde particles of MBNL with Kif1bα (left) and Kif1c (right), whereas Kif1bβ particles lack MBNL (center); time (sec) indicated in top or bottom right. Scale bar = 5 µm. Kymographs are displayed below still frames obtained from movies. White arrows denote co-transport events. Anterograde transport is shown from left to right. Scale bar = 10 µm. **f** Fraction of mScarlet-I-kinesin granules showing co-transport with EGFP-MBNL1; across 4 biological replicates. Bars represent mean. *p < 0.05, **p < 0.01 by one-way ANOVA with Tukey’s post-hoc test. **g** Western blots against RFP, MBNL1, and GAPDH following co-immunoprecipitation of mScarlet-Kif fusions with quantitation normalized to input MBNL1-EGFP protein; across 3 biological replicates. Lysates were collected after transient expression in N2A cells. Bars denote mean. **p < 0.01, ***<0.001 by one-way ANOVA with Tukey’s post-hoc test. Source data provided as a source data file.

We sought to identify putative kinesins that might transport MBNL1 by implementing a split kinesin recruitment assay in which mechanisms of cargo transport can be interrogated by linking various kinesin tails to a kinesin motor or dynein adapter^39,40^. Fusion of the dynein adapter bicaudal D2 (BicD2) to FKBP and kinesin tails to FRB facilitates dimerization and centrosome recruitment upon the addition of rapalog (Fig. 2a). We confirmed functionality of this system by co-expressing GFP-tagged transferrin receptor (TfR), tdTomato-tagged BicD2-FKBP (BicD2-FKBP), and Myc-tagged Kif1a-FRB or Kif13b-FRB in Neuro2A (N2A) cells. Only the Kif13b tail, and not the Kif1a tail, was able to recruit TfR to the centrosome, consistent with the established ability of Kif13b to carry TfR^39^ (Supplementary Fig. 2a). To confirm centrosome targeting, we visualized recruitment of TfR and Kif13b-FRB with BicD2-FKBP, coincident with γ-tubulin staining (Supplementary Fig. 2b). Using this assay, we screened 12 distinct kinesin tails (Kif1a, Kif1bα, Kif1bβ, Kif3a, Kif3b, Kif5a, Kif5b, Kif5c, Kif13a, Kif13b, Kif17, and Kif21b) for their ability to recruit EGFP-MBNL1. Only Kif1bα (and not Kif1bβ, an alternatively spliced isoform from the same gene) strongly recruited a cytoplasmic EGFP-MBNL1–40S isoform (Supplementary Fig. 2c) as well as both the 40 kDa and 41 kDa isoforms of MBNL1 (EGFP-MBNL1–40 and −41) (Fig. 2b, top panels). Recruitment was rapalog-dependent (Supplementary Fig. 2d) and specific to EGFP-MBNL1, but not other proteins such as SMN or TfR (Supplementary Fig. 2e, f). Swapping of the BicD2 adapter for the KIF1A motor domain resulted in rapalog-dependent recruitment of GFP-MBNL1 to plus ends of microtubules at the cell periphery, demonstrating that the association was tail domain-specific and not dependent on any specific motor domain (Fig. 2b, bottom panels; Supplementary Fig. 2g).

We subsequently simplified the centrosome recruitment assay by directly fusing FLAG-tagged BicD2 to Kif1bα and Kif1bβ tail domains (Fig. 2c). Using this approach, we also observed centrosomal targeting of EGFP-MBNL1–41 with Kif1bα but not Kif1bβ (Fig. 2d), thus validating this kinesin selectivity for MBNL1 with an independent assay. We additionally tested another kinesin tail that was not included in the initial screen, Kif1c. This kinesin shares ~58% sequence homology with the Kif1bα tail domain and is highly expressed in the central nervous system and muscle. We developed a quantitative assay to measure the extent of centrosome recruitment (Supplementary Fig. 3) and showed that Kif1bα and Kif1c (log_2_ mean centrosome enrichment: 0.506 vs. 0.560, Fig. 2d) recruit EGFP-MBNL1 to the centrosome more strongly than Kif1bβ (log_2_ mean centrosome enrichment: 0.149, ****p < 0.0001 by two-tailed Mann–Whitney *U* test, Fig. 2d).

The centrosome recruitment assay decouples kinesin tails from their motor domains and is not designed to assess co-transport of Kifs and MBNL1. To investigate this further, we performed IF on 12 DIV neurons transduced with AAV expressing HA-MBNL1–41 (Supplementary Fig. 4a). 3D quantitative colocalization analysis of select neurites supported previous findings: endogenous Kif1bα and Kif1c both displayed significantly higher levels of colocalization with HA-MBNL1 than endogenous Kif1bβ (Supplementary Fig. 4b, c). We subsequently investigated real-time co-transport by live cell imaging of EGFP-MBNL1–41 and mScarlet-I-tagged Kif1bα, Kif1c or Kif1bβ in DIV 8 primary cortical neurons. Approximately 50–60% of mScarlet-I-Kif1bα and mScarlet-I-Kif1c proteins showed processive runs (>2 µm) with EGFP-MBNL1 (*p < 0.05 by two-tailed Mann–Whitney *U* test, Fig. 2e, f), while <10% of mScarlet-I-Kif1bβ proteins showed co-transport (Fig. 2e, f). To confirm these observations biochemically, we co-expressed mScarlet-Kif proteins with EGFP-MBNL1-41 in N2A cells and performed co-immunoprecipitations using an RFP-Trap nanobody. EGFP-MBNL1 showed ~10-fold enrichment in the presence of Kif1bα and Kif1c as compared to Kif1bβ, which showed less than two-fold enrichment relative to mScarlet alone, as assessed by western blot (**p < 0.01, ***p < 0.001 by one-way ANOVA with Tukey’s test, Fig. 2g). Finally, an antibody against endogenous MBNL1 was able to co-immunoprecipitate endogenous Kif1c in N2A cells (Supplementary Fig. 4d). In summary, multiple experimental approaches reveal a selective and functional association between MBNL1 and specific kinesin tails.

### Expression of repeats and dominant negative kinesin tails causes mis-localization of MBNL-targeted mRNAs

**Fig. 3:**
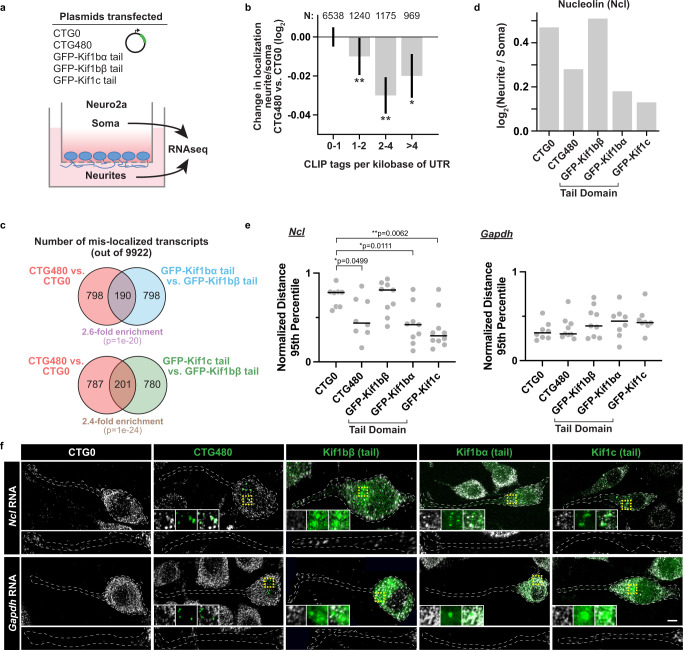
Depletion of MBNL or expression of kinesin tails leads to mis-localization of RNA targets. **a** Experimental design used to deplete MBNLs or over-express kinesin tail dominant negatives in differentiated N2A cells, followed by fractionation of soma and neurites. **b** Bars showing change in localization of mRNAs from neurite to soma in N2A expressing CTG480 as compared to CTG0, as a function of CLIP tag density per kilobase of 5′ and 3′ UTR. *p < 0.01, **p < 0.005 by two-tailed rank-sum test. Data represented as median values ± SEM. Data comes from one biological replicate. **c** Venn diagram showing overlap of mRNA targets mis-localized from neurite to soma upon perturbation by CTG480 relative to CTG0 or Kif1bα/Kif1c tails relative to Kif1bβ tail. *P* values computed by two-tailed Fisher’s Exact test, and enrichment of overlap was computed assuming independence between conditions. **d** The ratio of nucleolin mRNA between neurite and soma across conditions, as assessed by RNAseq. **e** Distances of *Ncl* and *Gapdh* HCR FISH spots from the soma of each cell across conditions. Distances were normalized as a fraction of total neurite length, and the 95th percentile of distances within each cell is plotted as an individual gray circle. Each cell (Ncl - CTG0: *n* = 8 cells, CTG480: *n* = 8 cells, Kif1bβ: *n* = 9 cells, Kif1bα: *n* = 9 cells, Kif1c: *n* = 10 cells; Gapdh - CTG0: *n* = 8 cells, CTG480: *n* = 9 cells, Kif1bβ: *n* = 9 cells, Kif1bα: *n* = 8 cells, Kif1c: *n* = 8 cells; across 3 biological replicates) contained >150 *Ncl* spots and >300 *Gapdh* spots. Bars represent the median across cells. *p < 0.05, **p < 0.01 by two-tailed Mann–Whitney U test. **f** Representative HCR FISH images for *Ncl* and *Gapdh* (red) with CTG repeats or dominant negative kinesin tails (green) in differentiated N2A cells. Scale bar = 10 µm. Source data provided as a source data file.

We hypothesized that either MBNL1 depletion or kinesin motor perturbation will have significant impacts on mRNA localization. We used a neurite fractionation assay to measure mRNA localization by plating N2A cells onto semi-permeable (1 µm-sized pores) membranes to facilitate neurite growth and separation. Plasmids expressing 480 CTG repeats or dominant negative kinesin tails with no motor domains^43^ were transiently introduced into N2A cells to functionally deplete MBNL activity or kinesin-dependent transport, respectively (Fig. 3a). Cell body and neurite RNAs were profiled by RNA-seq (TPMs for all transcripts in Supplementary Data 1). mRNAs containing a greater number of MBNL1 CLIP tags/kilobase in their UTRs, as previously determined^26^, showed stronger shifts away from neurites relative to soma in cells transfected with 480 CTG repeats as compared to control cells transfected with a DMPK fragment encoding 0 CTG repeats (*p < 0.01, **p < 0.005 by two-tailed rank-sum test, Fig. 3b, Supplementary Data 2). These results mimic previous observations of shifts in MBNL target mRNAs away from neurites following MBNL knockdown^32^, but uses CTG repeats to deplete MBNL activity rather than siRNA. Upon comparison to kinesin dominant negative conditions, a modest ~2.6-fold enrichment in overlap was observed between mRNAs mis-localized away from neurites after CTG480 expression (relative to CTG0) vs. mRNAs similarly mis-localized after Kif1bα tail over-expression (relative to Kif1bβ over-expression). A similar ~2.4-fold enrichment in overlap was observed when comparing mRNAs mis-localized after CTG480 expression to those mis-localized after Kif1c tail over-expression (Fig. 3c).

The mRNA encoding nucleolin (Ncl) was detected in neurites and markedly mis-localized after CTG480, Kif1bα tail, and Kif1c tail over-expression (Fig. 3d). Nucleolin, an RNA binding protein, inhibits axonal growth of sensory neurons through localization of importin β1 RNA and is present in axons, potentially playing a role to sense and regulate cell size^44^. MBNL1 CLIP tags and YGCY motifs are present in the UTRs and coding sequence of the *Ncl* transcript (Supplementary Fig. 5a), which prompted us to confirm subcellular localization by HCR RNA FISH. Indeed, *Ncl* mRNA spots were depleted from neurites after CTG480 over-expression, and occasionally co-localized with CTG repeats and dominant negative Kif1bα and Kif1c (Fig. 3f). Upon quantitation, the 95th percentile of *Ncl* HCR FISH spot distances (normalized as a percentage of total neurite length from the cell body centroid) was decreased after CTG480, Kif1bα tail, or Kif1c tail over-expression but not after CTG0 or Kif1bβ overexpression, while *Gapdh* localization was unaffected in all conditions (*p < 0.05, **p < 0.01 by two-tailed Mann–Whitney *U* test, Fig. 3e). *Ncl* mRNA mis-localization was assessed in another neuronal cell line, CAD, in response to the same perturbations. Similar mis-localization of *Ncl* mRNA was observed, in addition to co-localization with intranuclear repeat foci (Supplementary Fig. 5b, c). Together, these data suggest that MBNL1 and specific kinesins may play roles to localize mRNAs to neurites, and that localization of *Ncl* mRNA to neurites requires MBNL1 and Kif1bα or Kif1c.

### Association of MBNL1 with kinesin motors requires intact zinc fingers

**Fig. 4:**
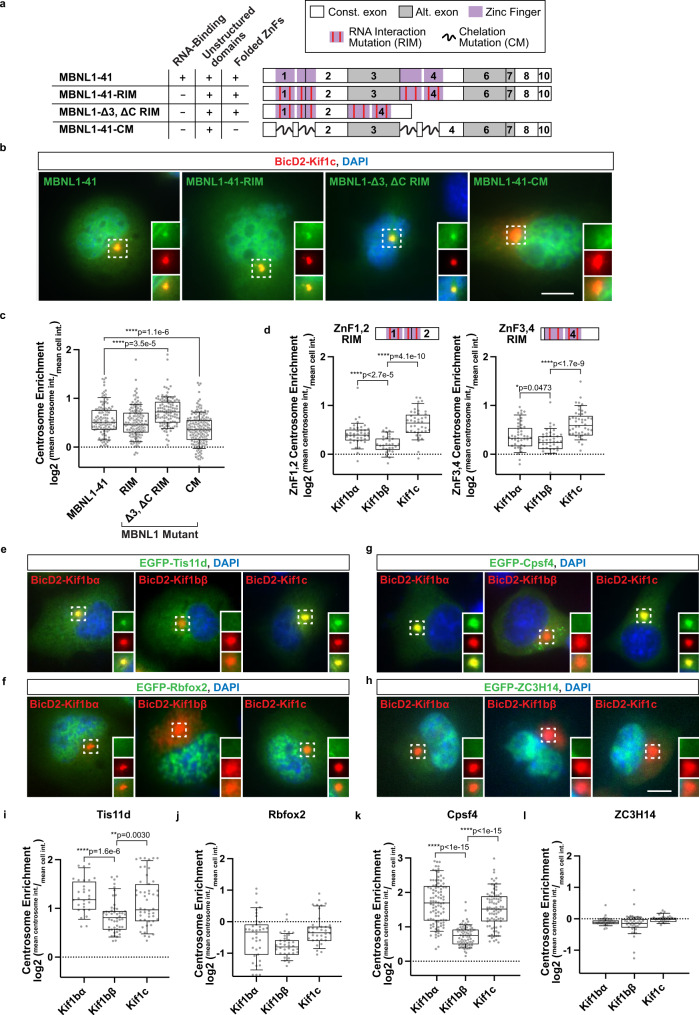
Zinc fingers of MBNL1 are necessary and sufficient to associate with kinesins in an RNA binding-independent manner, and structurally similar zinc fingers in other RBPs associate with the same kinesins. **a** Schematic of EGFP-MBNL1 mutants tested. **b** Representative images of centrosome enrichment for each mutant (green) as tested with BicD2-Kif1c tail fusions (red) in N2A cells. Nuclei labeled with DAPI (blue). **c** Quantitation of centrosome enrichment for each mutant. MBNL1-41: *n* = 112 cells (53, 19, and 40 cells in each replicate), MBNL1-41-RIM: *n* = 146 cells, MBNL1-Δ3, ΔC RIM: *n* = 106 cells, MBNL1-41-CM: *n* = 182 cells. **d** Quantitation of centrosome enrichment for EGFP-ZnF1/2 RIM and EGFP-ZnF3/4 RIM mutants using BicD2 kinesin tail fusions. MBNL1-ZnF1,2 RIM - Kif1bα: *n* = 39 cells, Kif1bβ: *n* = 29 cells, Kif1c: *n* = 40 cells; MBNL1-ZnF3,4 RIM - Kif1bα: *n* = 42 cells, Kif1bβ: *n* = 39 cells, Kif1c: *n* = 44 cells. **e** Representative images of centrosome enrichment for Tis11d (green); **f** Rbfox2 (green); **g** Cpsf4 (green); and (**h**) ZC3H14 (green) with BicD2 kinesin tail fusions (red) in N2A cells. **i** Centrosome enrichment quantitation for Tis11d - Kif1bα: *n* = 29 cells, Kif1bβ: *n* = 49 cells, Kif1c: *n* = 49 cells; and (**j**), Rbfox2 - Kif1bα: *n* = 36 cells, Kif1bβ: *n* = 35 cells, Kif1c: *n* = 36 cells; and (**k**), Cpsf4 - Kif1bα: *n* = 95 cells, Kif1bβ: *n* = 88 cells, Kif1c: *n* = 81 cells; and (**l**), ZC3H14 - Kif1bα: *n* = 43 cells, Kif1bβ: *n* = 40 cells, Kif1c: *n* = 25 cells. Bars represent median, box outline represent upper and lower quartiles, whiskers represent 10th and 90th percentiles. Dotted line represents no enrichment (=1). Scale bars = 5 µm. ****p < 0.0001, **p < 0.01, *p < 0.05 by two-tailed Mann–Whitney *U* test. Nuclei labeled with DAPI (blue). All experiments performed across 3 biological replicates. Source data provided as a source data file.

**Fig. 5:**
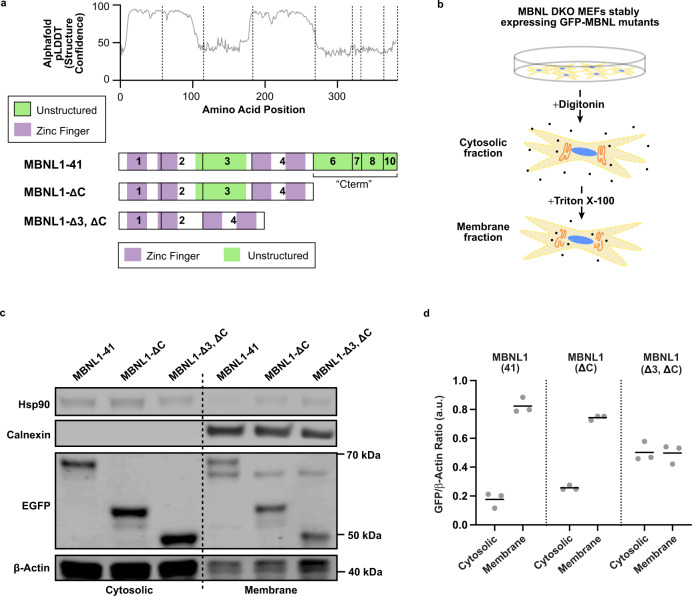
Unstructured domains of MBNL1 confer membrane association properties. **a** Structure confidence as assessed by Alphafold for the 41 kDa isoform of MBNL1; this metric is aligned to the exon structure below, and additional MBNL1 mutants which lack exon 3 and/or the C-terminal tail are also illustrated. **b** Schematic of detergent-based subcellular fractionation approach in Mbnl1/2 double knockout mouse embryonic fibroblasts. **c** Representative Western blot of EGFP-MBNL1 mutants following subcellular fractionation of cytosolic and membrane-associated compartments. Hsp90 shows enrichment in the cytosolic fraction; Calnexin the membrane fraction. **d** Quantitation of EGFP-MBNL1 concentration in each compartment as normalized to β-actin loading control, across 3 biological replicates. Source data provided as a source data file.

We sought to identify specific domains of MBNL1 responsible for kinesin association. We first assessed RNA dependency by employing RNA Interaction Mutants (RIMs) that disrupt RNA binding activity of MBNL1 zinc fingers (ZnFs) yet preserve ZnF structure^45^. Co-expression of EGFP-tagged MBNL1-41-RIM with FLAG-tagged BicD2-Kif1c yielded a similar level of centrosome enrichment as compared to wild-type EGFP-MBNL1-41, suggesting that kinesin association does not require RNA binding activity (log_2_ mean centrosome enrichment: 0.556 vs. 0.497, Fig. 4a–c). The non-ZnF domains of MBNL1 lack defined structure as predicted by AlphaFold^46,47^ (Fig. 5a, top), and roles of unstructured domains in other RBPs have been implicated in protein-protein interactions, liquid-liquid phase separation, and stress granule formation, among other functions^48^. The exon 3 linker between ZnF pairs confers flexibility for enhanced RNA binding and splicing regulation^49^. While the C-terminal tail was initially proposed to contain a trans-membrane domain^50^, it also contains a bipartite NLS sequence within exon 6 and a dimerization domain within exon 7^49,51^; presence of this C-terminal tail also enhances formation of stable CUG repeat foci^52^. To test the ability of unstructured domains to mediate kinesin association, we generated a mutant lacking both exon 3 and the C-terminal tail (MBNL1-Δ3, ΔC RIM) and co-expressed them with BicD2-Kif1c. This protein exhibited a more cytoplasmic distribution compared to the 41 kDa isoforms and, notably, an even stronger enrichment at centrosomes, potentially explained by its increased abundance in the cytoplasm (log_2_ mean centrosome enrichment: 0.724, ****p < 0.0001 by two-tailed Mann–Whitney *U* test, Fig. 4a–c). Therefore, the unstructured domains appear dispensable for kinesin-dependent transport.

Because the unstructured domains did not mediate kinesin association, we finally tested whether the ZnFs themselves possess these functions. We mutated several cysteine and histidine residues to prevent zinc ion chelation and thus induce mis-folding (CM, chelation mutants)^45^. EGFP-tagged MBNL1-41-CM showed significantly decreased recruitment at centrosomes when co-expressed with BicD2-Kif1c, revealing that, indeed, the ZnF domains are important for MBNL-kinesin interactions (log_2_ mean centrosome enrichment: 0.356, ****p < 0.0001 by two-tailed Mann–Whitney *U* test, Fig. 4a–c). Isolated ZnF pairs (ZnF1,2 and ZnF3,4) alone showed selective recruitment by BicD2-Kif1bα and BicD2-Kif1c relative to BicD2-Kif1bβ, although recruitment with Kif1c was slightly stronger (*p < 0.05, ****p < 0.0001 by two-tailed Mann–Whitney U test, Fig. 4d).

These observations led us to ask whether similarly structured ZnFs might also associate with the same kinesin tails. Tis11d, which regulates stability of AU-rich mRNAs, has ZnFs that fold similarly to those of MBNL^53^; therefore, we tested Tis11d in our centrosome recruitment assay. Strikingly, Kif1c and Kif1bα, but not Kif1bβ, efficiently recruited Tis11d (log_2_ mean centrosome recruitment Kif1bα vs. Kif1bβ vs. Kif1c – 1.235 vs. 0.826 vs. 1.131, **p < 0.01, ****p < 0.0001 by two-tailed Mann–Whitney *U* test, Fig. 4e, i). This pattern of recruitment was not observed with Rbfox2, which contains 3 RNA recognition motifs (RRMs) rather than ZnFs; this RBP exhibited little association with any of the 3 kinesin tails tested (Fig. 4f, j). Furthermore, Cpsf4, which recognizes polyadenylation sites in pre-mRNA and also contains similarly folded ZnFs, also showed selectivity for Kif1c and Kif1bα (log_2_ mean centrosome recruitment Kif1bα vs. Kif1bβ vs. Kif1c – 1.708 vs. 0.733 vs. 1.522, ****p < 0.0001 by two-tailed Mann–Whitney *U* test, Fig. 4g, k). However, ZC3H14, an RBP that contains C_3_H ZnFs with cysteine and histidine residues spaced differently from MBNL1, showed no centrosome recruitment with any of these 3 tails (Fig. 4h, l). Together, these findings reveal an association between MBNL1 ZnFs and specific kinesins for directed transport in an RNA binding-independent manner and also suggest a structural code underlying mRNA trafficking as controlled by specific RBP-kinesin associations.

### Unstructured domains of MBNL1 enhance association with membranes

We next addressed potential roles of the unstructured domains of MBNL. Previously, MBNLs were established to localize mRNAs to endoplasmic reticulum membranes for local translation of secreted proteins^26^. Here, observations of immobile granules in C2C12 myoblasts are consistent with a membrane anchoring function (Fig. 1f; Supplementary Movie 2). We directly tested whether unstructured domains of MBNL1 might facilitate membrane docking and association by studying EGFP-tagged, truncated versions of MBNL1 lacking either the C-terminal tail, or the C-terminal tail and exon 3 (ΔC or Δ3, ΔC mutants, Fig. 5a). These truncated MBNL1 proteins, along with full-length MBNL1, were stably introduced into mouse embryonic fibroblasts (MEFs) lacking endogenous Mbnl1 and Mbnl2, to preclude any potential piggybacking on target mRNAs that might localize to membranes via endogenous MBNL proteins. These cells were fractionated using digitonin and Triton X-100 to yield cytosolic and membrane-associated compartments, respectively (Fig. 5b). Successful separation was confirmed by enrichment of Hsp90 and calnexin in cytosolic and membrane fractions, respectively (Fig. 5c). All EGFP-tagged MBNL1 proteins were detected in both fractions, and quantitation of EGFP-MBNL1 relative to β-actin protein showed greatest membrane enrichment for full-length MBNL1, decreased enrichment upon removal of the C-terminal tail, and least enrichment upon removal of both C-terminal tail and exon 3 (Fig. 5d). These observations suggest that the unstructured domains of MBNL1 may contribute to membrane docking, with implications for mRNA localization and local translation.

### Recruitment of a synthetic mRNA to membranes by heterologous reconstitution of MBNL unstructured domains

**Fig. 6:**
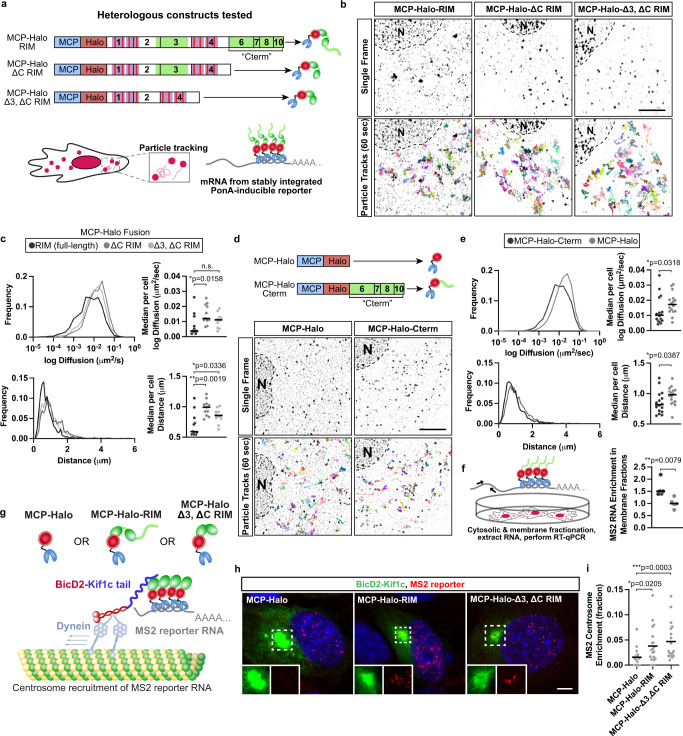
Heterologous reconstitution of domains from MBNL to localize RNAs in a kinesin- or membrane anchoring-dependent manner. **a** MS2 reporter system used to image single RNA molecules and reconstitute activities of MBNL domains to localize RNAs by fusion to MCP-Halo. Fusions generated include full-length MBNL1 or truncated MBNL1 without C-terminal tail and/or exon 3; ZnFs in these contexts were all RIM mutants incapable of binding RNA. **b** Particle tracks (multi-colored) from C2C12 myoblasts stably expressing MCP-Halo-RIM, MCP-Halo-ΔC RIM, or MCP-Halo-Δ3, ΔC RIM. **c** Inferred ensemble log diffusion coefficients (µm^2^/s) and maximum distance (µm) traveled for particles in each condition; MCP-Halo-RIM: 1233 particle tracks, MCP-Halo-ΔC RIM: 1039 particle tracks, MCP-Halo-Δ3, ΔC RIM: 934 particle tracks. Median per cell log diffusion coefficients (µm^2^/s) and maximum distance travelled for each particle (µm); *n* = 11 cells per condition across 3 biological replicates. **d** Schematic and particle tracks (multi-colored) from movies of C2C12 myoblasts stably expressing MCP-Halo or MCP-Halo-Cterm. **e** Inferred ensemble log diffusion coefficients (µm^2^/s) and maximum distance (µm) traveled for particles in each condition; MCP-Halo: 1205 particle tracks, MCP-Halo-Cterm: 885 particle tracks. Median per cell log diffusion coefficients (µm^2^/s) and maximum distance (µm); *n* = 16 cells per condition across 3 biological replicates. **f** Enrichment of MS2 reporter mRNA as assessed by RT-qPCR from membrane and cytosolic fractions of C2C12 myoblasts in the presence of MCP-Halo or MCP-Halo-Cterm, *n* = 5 biological replicates for each condition. **g** Schematic of MS2 reporter system used to reconstitute kinesin-dependent transport of RNA via MBNL1 zinc fingers. **h** Representative images from C2C12 myoblasts of HCR FISH (red) against the MS2 mRNA reporter in the presence of BicD2-Kif1c (green) and Halo-MCP fusions. Nuclei labeled with DAPI (blue). **i** Quantitation of the fraction of SunTag-MS2 RNA molecules present at the centrosome relative to the whole cell across each condition. Scale bars = 5 µm. *p < 0.05, **p < 0.01, ***p < 0.001 by two-tailed Mann–Whitney *U* test. Source data provided as a source data file.

While experiments above support association of MBNL unstructured domains with membranes, they do not prove a direct role in RNA targeting. To test this possibility, we developed stable C2C12 myoblasts expressing a ponasterone-inducible 45xMS2 RNA reporter, and also stably integrated various tetracycline-inducible fusions of MBNL1 domains to MS2 coat protein (MCP) and HaloTag (MCP-Halo) for live cell imaging of RNP granules^54^ (Fig. 6a). C2C12 myoblasts expressing MCP-Halo fusions without MS2 RNA reporter exhibit diffuse signal, whereas co-expression of the MS2 reporter yields visible motile granules in the cytoplasm (Supplementary Movie 5). The utility of this approach, which we term RBP Module Recruitment and Imaging (RBP-MRI), lies in the fact that each visible particle contains multiple copies of the RBP of interest, thus providing a readout biased toward behavior of that RBP. We obtained time lapse images of C2C12 myoblasts at 10 frames per second for 1 min and analyzed particle tracks using the Fiji plugin TrackMate^55^ (representative tracks shown in Fig. 6b; Supplementary Movie 3).

To assess contributions of the unstructured C-terminal tail and/or exon 3 to RNP dynamics, MCP-Halo-MBNL1-RIM was first compared to MCP-Halo-MBNL1-ΔC RIM and MCP-Halo-MBNL-Δ3, ΔC RIM (Fig. 6a, top). The use of RNA Interaction RIM mutants and the MS2-MCP system decouples observations of MBNL1-mediated RNA localization dynamics from RNA binding activities of MBNL1 ZnFs (Supplementary Movie 4). Analyses of ensemble particle diffusion coefficients and maximum distance traveled showed that particles containing the MBNL1 C-terminal tail travel shorter distances and are least diffusive (median ensemble diffusion: 6.7 × 10^−3^ μm^2^/s, median ensemble distance: 0.689 μm, Fig. 6c). The presence of exon 3 did not appreciably modulate particle behavior beyond the contribution of the C-terminus (median ensemble diffusion of ΔC RIM: 1.47 × 10^−2^ μm^2^/s, Δ3, ΔC RIM: 1.16 × 10^−2^ μm^2^/s; median ensemble distance of ΔC RIM: 0.988 μm, Δ3, ΔC RIM: 0.879 μm, Fig. 6c). Significant differences in per cell diffusion and maximum distance recapitulated ensemble observations (n.s. = *p* > 0.05, *p < 0.05, **p < 0.01 by two-tailed Mann–Whitney U test, Fig. 6c). We next tested whether the C-terminal tail alone, decoupled from any ZnFs, might be sufficient to enhance anchoring dynamics (Fig. 6d). MCP-Halo alone showed diffusive, Brownian motion, but MCP-Halo fused to the C-terminal tail showed much more confined dynamics (median ensemble diffusion of +C-term: 1.10 × 10^−2^ μm^2^/s, MCP-Halo: 2.31 × 10^−2^ μm^2^/s; median ensemble distance of +C-term: 1.15 μm, MCP-Halo: 1.55 μm, Fig. 6e). Again, we observed significant differences in per cell diffusion and maximum distance when the C-terminal tail is present (*p < 0.05 by two-tailed Mann–Whitney U test, Fig. 6e). Taken together, these results show that the MBNL1 C-terminal domain is necessary and sufficient for restricted mobility and RNP anchoring behavior.

To directly link these differences in mobility to membrane association of target RNAs, we performed subcellular fractionation, similar to experiments in Fig. 5, and assessed the role of the MBNL1 C-terminal domain to influence MS2 RNA abundance in membrane and cytosolic compartments using RT-qPCR (Fig. 6f). The presence of the C-terminal domain promoted ~60% greater association of MS2 reporter RNA with the Triton-sensitive membrane fraction relative to the cytosol, as compared to a Gapdh control mRNA (**p < 0.01 by two-tailed Mann–Whitney *U* test, Fig. 6f). These observations suggest that the C-terminal tail anchors MBNL1 and its target mRNAs to membranes, consistent with slower RNP dynamics as visualized in live cells. Analogous to the role of zinc fingers to mediate kinesin motor association, the C-terminal domain performs these functions in an RNA binding-independent manner.

### Kinesin tail-dependent transport of a synthetic mRNA reporter by heterologous reconstitution of MBNL ZnFs

In previous experiments, we showed that MBNL1 ZnFs associate with specific kinesin tails, as supported by co-transport in cells and by co-immunoprecipitation in a detergent-resistant manner. However, this does not prove that these associations facilitate RNA transport. To demonstrate this, we again turned to our RBP-MRI approach. To assess whether MBNL ZnFs can localize RNAs in a kinesin-dependent manner, we co-expressed a BicD2-Kif1c tail fusion, which pulls cargoes to the centrosome, with stably integrated tet-inducible MCP-Halo, MCP-Halo-MBNL1 (RIM), or MCP-Halo-MBNL1 (Δ3, ΔC RIM) fusions (Fig. 6g). We performed HCR FISH against the MS2 reporter RNA and counted RNA molecules at the centrosome and in the entire cell (Fig. 6h). Two to three-fold greater MS2 RNA molecules localized to the centrosome in the presence of both MCP-Halo-RIM fusions, as compared to MCP-Halo alone (*p < 0.05, ***p < 0.001 by two-tailed Mann–Whitney U test, Fig. 6i). These observations suggest that MBNL ZnFs are sufficient to localize RNAs to distinct subcellular locations in a kinesin-dependent manner, independent of ZnF RNA binding activity. Overall, this heterologous system allowed us to decouple and characterize distinct activities of modular MBNL protein domains then synthetically reconstitute them in a variety of systems.

### MBNLs regulate the biogenesis of their kinesin transport partners

**Fig. 7:**
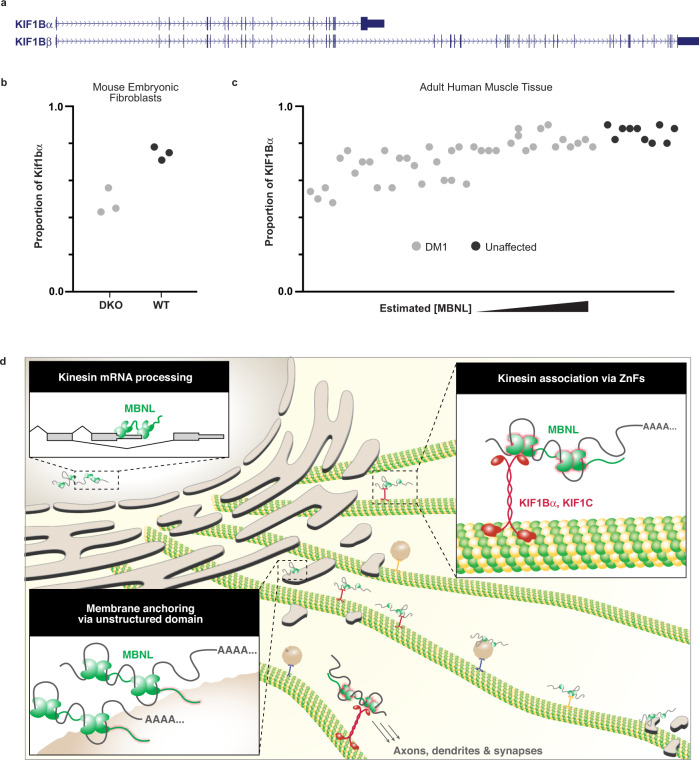
MBNL1 regulates the production of kinesin tails with which it associates in the cytoplasm. **a** Representation of alternative last exon usage in the KIF1B gene, which yields two isoforms. **b** Proportion of Kif1bα vs. total Kif1b in Mbnl1/2 double knockout mouse embryonic fibroblasts and (**c**), KIF1Bα vs. total KIF1B in human DM1 tibialis. **d** Model for how MBNLs regulate production of kinesin tails with which they associate in the cytoplasm to travel along microtubules and anchor at membrane destinations.

Importantly, Kif1bα and Kif1bβ are alternative isoforms generated from the *Kif1b* gene; Kif1bα is generated by use of an alternative last exon and earlier polyadenylation site relative to Kif1bβ (Fig. 7a). MBNL is well-established to regulate alternative splicing, alternative last exon usage, and alternative polyadenylation in the nucleus. To our surprise, examination of previously generated datasets^22,56^ show that the ratio of Kif1bα to total Kif1b changes upon MBNL depletion. Overall Kif1bα/KIF1Bα levels relative to total Kif1b/KIF1B are decreased in mouse embryonic fibroblasts lacking Mbnl1 and Mbnl2 (DKO), and in human DM1 tibialis anterior biopsies (Fig. 7b, c). Consistent with a potential direct role in enhancing the cleavage and polyadenylation of Kif1bα, MBNL CLIP tags are found in the 3′ UTR of this isoform^26,27^. These findings suggest that MBNL regulates the production of specific kinesin isoforms with which it subsequently associates in the cytoplasm. Thus, we find that not only does MBNL mediate several distinct steps in RNA localization, including RNA binding, kinesin engagement, and membrane anchoring, but that it also regulates the abundance of the specific motors with which it interacts by alternative splicing (Fig. 7d). In principle, this molecular circuitry may allow the cell to appropriately match supply and demand of a specific motor by using the same RBP in both the nucleus and cytoplasm.

## Discussion

Highly differentiated cells use molecular motors and RBPs to localize mRNAs to subcellular compartments to generate and maintain local protein gradients. This is particularly apparent in neurons^57^, muscle^58,59^, heart^60^, intestinal epithelium^61^, and other cell types, but specific interactions between molecules necessary for mRNA localization are not clear. Immunoprecipitation-based methods have identified putative binding partners for a number of kinesins, most notably the conventional kinesin-1, kinesin-2, and kinesin-3 family members and additional kinesins such as KIF21, KIF26A, and KIF13B^43,62–66^. Other unbiased approaches^67^ or yeast 2-hybrid systems^68^ have been taken to identify RBP-kinesin interactions at higher throughput scale. FMRP, ZBP1, hnRNP Q, Pur-α, SFPQ, Staufen1, egalitarian, and tropomyosin are RBPs that have been associated with specific kinesins in mammalian and *Drosophila* systems, but the sheer number of motors and cargoes implies that we have only explored a small subset of all possible interactions^4,7–9,69–75^. Here, we show that MBNL interacts with specific motor proteins, Kif1bα and Kif1c, for directed transport (Fig. 2). No RBPs were previously known to specifically interact with these motors. Surprisingly, we found that these interactions are mediated through ZnF domains in a non-RNA binding-dependent manner, with each pair of ZnFs sufficient for kinesin association (Fig. 4b–d). Similarly structured ZnFs, such as Tis11d and Cpsf4, showed similar kinesin selectivity. Additional studies would be required to determine the precise nature of kinesin-MBNL association, and whether additional protein adapters are required to form, stabilize, or disrupt these interactions. Overall, our findings raise the possibility of a broader, well-defined code that dictates RBP-kinesin specificity, with implications for understanding and manipulating microtubule-dependent RNA localization patterns.

Kif1bα has been implicated in mitochondrial trafficking^76,77^ and localizes to postsynaptic densities^78^, but was not known to localize RNAs or associate with RBPs. The alternatively spliced isoform Kif1bβ has been more extensively studied; it transports synaptic vesicle precursors through IGF1R, regulates axonal outgrowth, and is implicated in Charcot-Marie-Tooth disease and neuroblastoma^14,79^. Kif1c, whose tail domain is 58% similar to Kif1bα (compared with 46% similarity to Kif1bβ and 21% similarity to Kif5b), is involved in transport of mitochondria and dense core vesicles, and is a fast kinesin^67,80^. Although it is plus-end-directed, it undergoes bidirectional transport through association with dynein and is subject to an autoinhibitory mechanism in which its stalk inhibits the motor domain in the absence of Hook3 and Ptpn21^81,82^. Kif1c transports mRNAs, including its own transcript, to cell protrusions through an APC-dependent mechanism^83,84^. Notably, at the transcript level, Kif1bα and Kif1c together comprise ~75% and ~50% of the total kinesin transcript pool in muscle and heart tissue, respectively, and ~26% and ~30% in the substantia nigra and spinal cord, respectively (Supplementary Data 3). These high abundances might imply particularly important roles for these motors to transport cargoes in these tissues.

From these studies, it is unclear whether a single ZnF pair can simultaneously associate with binding targets. Differential RNA binding properties of each ZnF pair have been characterized, with ZnF(1,2) showing greater specificity than ZnF(3,4) for YGCY motifs^38^. In our assays, we did not detect strong differences in kinesin association between ZnF(1,2) vs. ZnF(3,4) (Fig. 4d). Thus, one possibility could be that one ZnF pair engages kinesin, while the other pair engages RNA. Alternatively, both pairs of ZnFs could engage RNA simultaneously with a single kinesin tail, or potentially even 2 separate kinesin tails or motors. Given the dimerization potential of MBNL1 via exon 7^49^, multivalency for both RNA binding and kinesin association could be achieved, with a total of 4 ZnF pairs linked together in a single complex. We observed accumulation of *Ncl* mRNA, an MBNL target, at CUG RNA foci in both N2A and CAD cells (Fig. 3f, Supplementary Fig. 5b, c), raising the possibility that multiple RNAs could be tethered through 2 distinct MBNL ZnF pairs, or via dimerized MBNL proteins. Indeed, expression of MBNL lacking the C-terminal tail lessens the stability of CUG RNA foci^52^. Given that kinesin associates with MBNL ZnFs, the potential for tethering may not only apply to RNAs but also motors—since kinesins and dyneins work in teams to carry protein complexes and/or vesicles^85,86^, RBPs or RNPs could serve as multivalent adapters to bring multiple motors together into a single complex.

RNA localization has been proposed to involve not only RNP assembly and engagement with motors, but also anchoring to destinations. The sushi-belt model proposes that “hungry synapses” remove RNPs from the belt when activated^87^. It is unclear how RNAs anchor at synapses, although interactions with intracellular membranes, including ER, is a possibility. MBNL targets are enriched in signal peptides, with many encoding membrane proteins^26^. ER is enriched at dendritic branch points and synapses^88^, which may also serve as sites of docking for MBNL-containing RNPs. We found that the MBNL1 C-terminal domain alone is sufficient to slow RNP movement, limit total distance traveled, and enhance association of a reporter RNA to triton-sensitive fractions of mouse myoblasts. Interestingly, a paralog of Tis11d, Tis11b, helps form an ER-associated membraneless domain^89^. Therefore, not only do MBNL and Tis11 family members share preferences for certain kinesins via their ZnFs, but they may also exhibit parallels in membrane anchoring activities.

The association of RBPs with membranes also has implications for RNA localization by way of motile endosomes and lysosomes. Tethering of RNA granules to endosomes in a Rab7a-dependent manner^11^ or to lysosomes in an ANXA11-dependent manner^12^ may hold implications for MBNL-mediated anchoring of RNAs to membranes. Similar to MBNL, multiple domains of ANXA11 perform distinct functions; the low-complexity domain mediates phase separation with membraneless RNA stress granules and the C-terminal annexin-repeat region acts as a binding partner for lysosomes. Mutations in the low-complexity domain and annexin domains of ANXA11 have also been associated with ALS, highlighting the importance of unstructured domains and membrane tethering for cellular function and homeostasis^90,91^. It remains to be determined whether MBNL also tethers RNAs to endosomes or lysosomes, and if there are preferences according to Rab composition. MBNL is also post-translationally modified by ubiquitination^35^, however, it is unknown whether this or other modifications impact MBNL-membrane associations. Together, these observations highlight the modularity of both globular and unstructured domains of RBPs to carry out post-transcriptional regulation, and the potential for multiple overlapping mechanisms to co-exist in the cell.

Several neurological, neurodegenerative, and neuromuscular diseases are caused by mutations or perturbations to RBPs and kinesins. Mutations in highly expressed RBPs such as hnRNP A1, hnRNP A2B1, TDP-43, and FUS are associated with ALS^92^. Mutations in the ATP binding site of KIF1Bβ are linked to Charcot-Marie-Tooth disease type 2A^14^. Motor or tail domain mutations in KIF1C cause autosomal recessive hereditary spastic paraplegia or ataxia^93,94^. Motor or stalk domain mutations in KIF5A cause hereditary spastic paraplegia, and tail domain mutations in KIF5A cause ALS^17,18,92^, which may be explained by disrupted auto-inhibition^95^. Depletion of SMN in spinal muscular atrophy perturbs RNP granule formation and has deleterious effects on local translation in motor neurons^2,96^. In DM1, MBNLs are depleted by expanded CTG repeat expression, with clear changes in global splicing patterns and potential changes in RNA localization in myoblasts^26^. Our observations here demonstrate that expression of expanded CTG repeats impairs RNA localization in neural cells. Taken together, these findings prompt considerations about other diseases that could be exacerbated by compromised transport, including Huntington’s disease, Alzheimer’s disease, and other dementias. Here, we explored functional consequences of MBNL sequestration and kinesin perturbation by dominant negative tail expression in N2A and CAD neurites. As might be expected, we found modest overlap in shared targets of MBNL, Kif1bα, and Kif1c, given that these kinesins likely also associate with many other RBPs. Nucleolin mRNA was confirmed as a bona fide target that relies on these players; it encodes an RBP that regulates ribosome biogenesis but also localizes to distal neurites^44,97,98^. Ncl protein anterogradely transports importin β1 mRNA to neurites and associates with Kif5a through its glycine/arginine-rich domain^44,99^. Locally translated importin β1, together with Ncl, is brought back to the soma by retrograde transport to control cell size. Here, we find that localization of Ncl mRNA itself is influenced by MBNL and Kif1bα/Kif1c, raising the possibility that Ncl protein in neurites may arise via local translation. Indeed, MBNL1 CLIP tags appear throughout the Ncl mRNA, and it may be translationally repressed while transported by kinesins. Thus, this circuit may control cell size during neurogenesis and potentially even modulate susceptibility to cancer, as mutations connected to KIF1B have been implicated in neuroblastomas^100^, paragangliomas, and pheochromocytomas^101^.

The observation that MBNL protein activity influences the abundance of Kif1bα raises the possibility of feedback between nuclear and cytoplasmic processes, and indeed, the proportion of nuclear vs. cytoplasmic MBNL depends on cell type, state, and ubiquitination status^35^. It remains to be determined whether MBNL directly regulates the splicing or polyadenylation of Kif1b pre-mRNA, as suggested by MBNL-CLIP tags, but an attractive possibility is that this architecture allows a single RBP to control and buffer the number of transport vehicles it can employ to carry out its functions. During muscle differentiation, MBNL activity rises, Kif1bα abundance increases, and there is overall increased reliance on microtubules to distribute mRNAs throughout the cytoplasm^58^. Conversely, depletion of MBNLs in DM not only impairs localization functions of MBNLs, but also reduces the total pool of Kif1bα available for transport. MBNL CLIP tags also appear in the Kif1c 3′ UTR, whose mRNA is also localized to neurites and has multiple alternative polyadenylation sites^27,83^. An open question is whether MBNL may also regulate Kif1c RNA processing and local translation, thus controlling expression of an additional transport partner. Finally, although KIF13A did not recruit MBNL in our kinesin tail screen, exon 32 is believed to be an MBNL splicing target and is a robust biomarker of disease status in DM1—functions of exon 32 are unknown, and a potentially fruitful line of investigation may be to probe its roles in the pathways discussed here^102,103^.

Finally, we establish methods for studying kinesin-RBP-mRNA interactions. Our adaptation of the centrosome recruitment assay could reveal many previously unknown kinesin-RBP interactions if applied to additional targets. RBP-MRI reveals RBP properties by directly tethering RBP domains to the MS2 coat protein and assembling them onto an MS2 reporter RNA, painting a “dynamic caricature” of the RBP. Our assay has similarities to the “TnT translation and tether” assay^104^, but uses Halo signal to directly image RNPs that contain the RBP of interest. Inducible components yield signal to noise properties required to generate high quality movies amenable to automated computational analyses and could be expanded to many additional RBPs. The techniques outlined here provide a platform for further investigation of kinesins, RBP domains, and mRNAs that influence transport, with implications for our global understanding of mRNA localization, as well as how they may go awry in genetic diseases, including DM.

## Methods

### Statistics and reproducibility

All experiments, with exception of neurite fractionation, were performed at least three times. Images shown in panels represent consistent results observed across all replicates. Statistical analyses performed are referenced in each figure legend. All analyses were performed using GraphPad Prism software (Version 9.3.1).

### Plasmids and cloning

All plasmid fusions assembled in-house were generated through standard ligation-based methods using the In-Fusion cloning kit according to manufacturer protocols (Takara, 638920). MBNL1 RNA-interaction and chelation mutant sequences were designed based on plasmids generated by Andrew Berglund^45^. Tis11d coding sequence was synthesized by IDT, Rbfox2 was amplified from mouse cDNA, Cpsf4 was amplified from mouse cDNA, and ZC3H14 was subcloned from a plasmid kindly gifted by Anita Corbitt (Emory University). All above sequences along with MBNL1 and MBNL2 isoforms imaged in primary neurons were cloned downstream of EGFP in a pEGFP-C1 plasmid backbone under control of a CMV promoter (Takara). Full-length kinesin sequences were amplified from mouse cDNA and inserted downstream of an mScarlet-I-containing plasmid in a pcDNA3.1 backbone under the control of CMV promoter and with a flexible glycine-serine linker following mScarlet-I.

For dominant negative conditions, kinesin tail sequences were amplified from mouse cDNA and cloned into pEGFP-C1 as described above. For the centrosome recruitment assay, tail sequences were inserted downstream of a BicD2 dynein adapter in pcDNA3.1 under control of a CMV promoter. Plasmids containing FRB split kinesin tails and FKBP motors were a gift from Gary Banker and described previously^39,40^. Plasmids carrying exons 11–15 of the DMPK gene expressing either 0 or 480 CTG repeats under the control of a CMV promoter were a gift from Tom Cooper (Baylor College of Medicine).

PB-Neo-pERV3 contained a bicistronic ecdysone receptor expression cassette under control of EF-1α promoter and PGK-driven neomycin resistance cassette was first stably introduced into C2C12 myoblasts. An MS2 reporter plasmid (PBPuroPonA-56xSunTag-45xMS2) containing a 56× SunTag coding sequence with a minimal intron upstream of 45x MS2 hairpins in the 3′ UTR and downstream of an ecdysone-responsive promoter with a blasticidin resistance cassette under control of a PGK promoter was then introduced. Finally, plasmids containing tandem MCP with nuclear localization sequences were cloned downstream of a tetracycline-responsive promoter with a puromycin resistance cassette under control of a PGK promoter. An MCP sequence was derived from a plasmid deposited by Robert Singer in the Addgene plasmid repository (Addgene, 40649). Subcloning into the MCP plasmid sequentially introduced the HaloTag sequence (Promega) and MBNL fusions (PB-PuroTet-MCP-HaloTag). MBNL1-EGFP isoform sequences and mutants were amplified from previously established plasmids (see above) and were designed for stable expression of nuclear and cytoplasmic MBNL1 in C2C12 myoblasts and mouse embryonic fibroblasts (MEFs). MS2 array sequence is derived from a plasmid gifted by Edouard Bertrand. All transgenes for inducible MS2-MCP system and stable expression of MBNL1-EGFP isoforms or mutants were flanked by PiggyBac transposon arms^105,106^. Full plasmid sequences have been provided as a data file (Supplementary Data 4).

### Cell lines and transfection

Neuro2a cells (N2A; gift from Chris Burge), MBNL double-knockout MEFs (DKO MEFs; gift from Maury Swanson), and C2C12 myoblasts (ATCC, CRL-1772) were cultured in DMEM supplemented with 100 U/mL penicillin, 100 mg/mL streptomycin, and 10% fetal bovine serum. CAD cells (gift from Chris Burge) were cultured in 1:1 DMEM:Ham’s F12 supplemented with 100 U/mL penicillin, 100 mg/mL streptomycin, and 8% fetal bovine serum. All cells were kept in a humidified incubator with 5% CO_2_ at 37 °C. To induce N2A and CAD cell differentiation, media was changed to DMEM without serum.

For the split kinesin assay, N2A cells were transfected with Lipofectamine 2000 (Thermo Fisher, 11668027) according to manufacturer’s instructions, then imaged after overnight expression and ~3 h incubation with 500 nM A/C heterodimerizer linker drug (Takara, 635055) or 100% ethanol. 10 μM S-Trityl-L-cysteine was added shortly after transfection for BicD2 experiments to stall cells in interphase and stabilize centrosomes. All other transfections in N2A, CAD, C2C12, and MEF lines were performed using TransIT X2 (Mirus, MIR6003) according to manufacturer’s instructions.

### Production of stable C2C12 myoblasts and MEFs

To make an MCP-MS2 reporter line, first, a wild-type C2C12 myoblast line was co-transfected with PB-Neo-pERV3 and an mPB plasmid expressing a PiggyBac transposase. PiggyBac allows for stable integration into the host cell genome^105^. C2C12 myoblasts were then selected with 500 µg/mL G418 (Geneticin) for 2 weeks. Following recovery, C2C12s were then transfected with PB-PuroPonA-56xSunTag-45xMS2 for stable integration and selected with 10 µg/mL blasticidin for 1 week. Following a second recovery, C2C12 myoblasts were transfected with PB-PuroTet-MCP-HaloTag alone or fused to various MBNL1 proteins for stable integration and selected with 5 µg/mL puromycin for 1 week.

Other C2C12 myoblasts expressing various MBNL1 isoforms or DKO MEFs expressing MBNL1-41 and truncated mutants in PiggyBac plasmids were transfected similar to above and selected with 5 µg/mL puromycin for 1 week. All transfections for stable cell lines were performed using TransIT X2 (Mirus, MIR6003) according to the manufacturer’s instructions.

### Primary mouse cortical neuron culture, transfection, and transduction

Cortices isolated from C57BL6 E17 mouse embryos were dissected and dissociated with trypsin at 37 °C (Thermo Fisher Scientific, 15050-065). Cells were plated in MEM/FBS on coverslips previously coated with 1 mg/ml poly-L-lysine in Borate Buffer for 72 h followed by three 1 h washes with sterile water. After 2 h, medium was replaced with astrocyte conditioned Neurobasal Plus Medium (Gibco, A3582901) supplemented with B-27 Plus Supplement (Gibco, A3582801) and GlutaMAX Supplement (Gibco, 35050061). Mouse care and embryonic neuron isolation were performed in adherence to policies and regulations from the Institutional Animal Care and Use Committee (IACUC) at Emory University.

For live imaging of EGFP-MBNL-kinesin co-transport in primary cortical neurons, cells were transfected with Lipofectamine 2000 (Thermo Fisher, 11668027) as follows: fresh culture medium was added to the cells 1:1, then half of the medium was collected. DNA/Lipofectamine mix was prepared according to manufacturer’s instructions and left with the cells for 1.5 h, after which medium was replaced with the collected medium. Neurons were imaged after overnight expression following incubation with 100 ng/mL BDNF for 30 min. Live cell imaging of EGFP-MBNL1 and EGFP-MBNL2 isoforms involved transfection with Neuromag (OZ Biosciences, NM50200) at 7 DIV according to the manufacturer’s protocol. Cells were incubated on a magnet at 37 °C for 20 min, incubated with 100 ng/mL BDNF after 18–24 h, then imaged after 30 min. 8 DIV cortical neurons were transduced with adeno-associated virus containing ssDNA encoding HA-MBNL1. Four days later, at 12 DIV, the cells were processed for quantitative colocalization imaging of HA-MBNL1 and endogenous KIFs.

### Immunofluorescence (IF)

Cells were washed once with phosphate-buffered saline (PBS) then fixed with 4% paraformaldehyde (PFA) for 10 min at room temperature (RT). Cells were then washed 3× with PBS and permeabilized with 0.2% Triton X-100. Cells were washed again 3× with PBS (or Tris-Glycine buffer for neuronal staining) and incubated with primary antibody diluted in PBS containing 3% bovine serum albumin (5% for neuronal staining) and 0.1% Tween-20 (AB buffer) for 1 h at RT. Primary antibodies used for IF include: mouse anti-MBNL1 (1:100, Developmental Studies Hybridoma Bank, MB1a(4A8)), mouse anti-MBNL2 (1:100, Developmental Studies Hybridoma Bank, MB2a(3B4)), rabbit anti-Map2 (1:1000, Millipore-Sigma, AB5622), guinea pig anti-Map2 (1:1000, Synaptic Systems, 188 004), anti-Tau (1:1000, Aves Labs, TAU), mouse anti-Myc (1:100, Developmental Studies Hybridoma Bank, 9E10), mouse anti-γ-Tubulin (1:500, Abcam, ab11316), rabbit anti-FLAG (1:1000, Cell Signaling Technologies, D6W5B), mouse anti-KIF1B - recognizes KIF1Bβ (1:50, EMD Millipore, MABC309), mouse anti-KIF1B - recognizes KIF1Bα (1:50, Santa Cruz, sc-376246), rabbit anti-KIF1C (1:200, Abcam, ab125903), rabbit anti-HA (1:800, Cell Signaling Technologies, C29F4), and mouse anti-HA (1:1000, BioLegend, 901501). Cells were then washed 3X with PBS and incubated with secondary antibody in AB buffer (anti-mouse Alexa Fluor 488, 1:500, Thermo Fisher, A11001; anti-mouse Alexa Fluor 647 1:500, Thermo Fisher, A21235; anti-rabbit Alexa Fluor 647 1:1000, Thermo Fisher, A32733; anti-chicken 633, 1:500, Thermo Fisher, A21103; anti-guinea pig 633, 1:500, Thermo Fisher, A21105) for 1.5 h at RT. Cells were washed 3X with PBS with DAPI (1 ng/uL) included on the final wash, mounted, and imaged.

### Western blotting

For MEFs cytosolic and membrane lysates, protein concentrations were quantified using Pierce BCA protein assay kit (Thermo Fisher, 23225), processed by diluting in 4X LDS buffer (Thermo Fisher, NP0008), and separated on 4–12% Bis-Tris polyacrylamide gels (Thermo Fisher, NP0336) after denaturation for 5 min at 95 °C. Gel electrophoresis was performed for ~90 min at 100 V in 1X MOPS running buffer (50 mM 3-(N-morpholino)propanesulfonic acid (MOPS), 50 mM Tris base pH 8.0, 3.5 mM SDS, 1 mM ethylenediaminetetraacetic acid (EDTA)). Transfer was performed on activated polyvinylidene fluoride (PVDF) membranes (Bio-Rad, 1620264) using iBlot 2 dry transfer machine (Thermo Fisher). Membranes were blocked with SEA BLOCK (Thermo Fisher, 37527) for 30 min at RT. Membranes were then incubated with primary antibodies diluted in SEA BLOCK overnight at 4 °C. Primary antibodies used for western blot include: mouse anti-Hsp90 (1:1000, Abcam, ab13492), rabbit anti-Calnexin (1:1000, Abcam, ab22595), chicken anti-GFP (1:5000, Abcam, ab13970), and rabbit anti-β-Actin (1:2000, Cell Signaling Technologies, 4967). The following day, primary solution was removed, and membranes were washed 2X with PBS and 0.1% Tween-20 (PBST). Membranes were then incubated with secondary antibodies (all from LICOR, 1:5000 dilution, IRDye 800CW donkey anti-rabbit, #926-32213; IRDye 800CW donkey anti-chicken #926-32218; IRDye 680LT donkey anti-mouse, #926-68072; IRDye 680LT donkey anti-rabbit, #926-68073) diluted in SEA BLOCK for 1 h at RT. Membranes were then washed again 2X with PBST and imaged using a LI-COR Odyssey CLx scanner at 700 and 800 nm excitation wavelengths.

### Immunoprecipitation

N2A cells were collected from 10 cm^2^ plates 48 h after transfection of EGFP-MBNL and kinesin plasmids and washed 2X with PBS. Cell pellets were resuspended with lysis buffer (10 mM Tris-HCl pH 7.5, 150 mM sodium chloride (NaCl), 0.5 mM EDTA, 0.5% NP-40) containing protease/phosphatase inhibitor. Protein concentration was determined using Pierce BCA Assay (Thermo Fisher) and equal amounts of protein were incubated with 25 uL RFP-Trap magnetic agarose rotating for 1 h at 4 °C (Proteintech, rta-10). The beads were washed three times and boiled in SDS sample buffer for 10 min at 95 °C.

For immunoprecipitation of endogenous MBNL1, a modified CLIP protocol was used (Wang et al., 2009). N2A cells, plated the day before, were scraped off with PBS and centrifuged at 500 *g* for 4 min. With the supernatant removed, each pellet was resuspended in 500 uL lysis buffer (50 mM Tris-HCl pH 7.5, 100 mM NaCl, 1 mM MgCl_2_, 0.1 mM CaCl_2_, 1% NP-40, 0.1% sodium deoxycholate, 0.05% SDS), containing protease/phosphatase inhibitor and endonuclease (Benzonase, Millipore Sigma, E8263). The resuspended pellet was incubated on ice for 30 min with extensive pipetting every 10 min. During this time, 100 uL of protein A Dynabeads (Invitrogen, 10001D) were transferred to a 1.5 mL microcentrifuge tube and washed 3X with lysis buffer. After the third wash, the beads were resuspended in 200 uL of lysis buffer and 3 ug of rabbit ɑ-MBNL1 antibody (Millipore Sigma, ABE241) or normal Rabbit IgG (Millipore Sigma, 12–370) was added. The antibody-bead mixture was then rotated at 4 °C for 30–60 min. Meanwhile, the cell lysates were centrifuged at maximum speed for 3 min, the supernatant was carefully collected, and the protein concentration was determined using Pierce BCA Assay (Thermo Fisher). The antibody-conjugated beads were washed again 3X with lysis buffer and an equal amount of protein was added to each. Samples were rotated overnight at 4 °C. The next day, the beads were washed two times with high-salt wash buffer (50 mM Tris-HCl pH 7.5, 1 M NaCl, 1 mM EDTA, 1% NP-40, 0.1% sodium deoxycholate) and boiled in SDS sample buffer for 5 min at 95 °C.

Immunoprecipitated proteins were separated on 4–20% Tris-Glycine polyacrylamide gradient gels (Bio-Rad, 4561096) and transferred onto 0.45 mm nitrocellulose membranes. Membranes were blocked in 5% BSA in PBS before being incubated overnight at 4 °C with rabbit ɑ-MBNL1 antibody (1:1000, Millipore Sigma), mouse ɑ-MBNL1 antibody (1:100, DHSB, 4A8), mouse ɑ-RFP antibody (1:1000, Proteintech, 5F8), rabbit ɑ-Kif1c (1:500, Proteintech, 12760-1-AP), or mouse ɑ-KIF1bβ (1:500, EMD Millipore, MABC309). The following day, membranes were incubated with IRDye 680LT donkey anti-mouse (1:20000, LICOR, #926-68072) and either IRDye 680LT donkey anti-rabbit (1:20000, LICOR, #926-68073) or HRP mouse anti-rabbit IgG light chain (1:5000, Jackson ImmunoResearch, 211-032-171) for 1 h at RT. All blots were also incubated with Rhodamine-conjugated ɑ-GAPDH human Fab fragment (1:1000, Bio-Rad, 12004168). HRP was detected using Tanon™ High-sig ECL Western Blotting Substrate (ABclonal, 180-5001) and the blots were imaged on the ChemiDoc MP system and quantified using Image Lab software (Bio-Rad).

### Hybridized chain reaction fluorescence in situ hybridization (HCR FISH)

Probes, amplifiers, and buffers for performing HCR FISH 3.0 were purchased from Molecular Instruments^107^. Probe sets constituted 10–30 probes, depending on target sequence. Cells were fixed with 4% PFA for 10 min at RT, washed 3X with PBS, then permeabilized with 0.2% Triton X-100. For combined IF/FISH experiments, cells were washed 3X with PBS, IF was performed first with 0.5% ultra-pure bovine serum albumin (Thermo Fisher, AM2616) and primary antibody in AB buffer for 1 h. After washing 3X with PBS, secondary antibody incubation in AB buffer was performed for 1.5 h followed by a second fixation in 4% PFA for 10 min at RT before proceeding with FISH. Cells were washed once with PBS then once with 2X saline-sodium citrate (SSC) and pre-hybridized with hybridization buffer (Molecular Instruments) for 30 min in a 37 °C humidified chamber. Primary probes were diluted in hybridization buffer at a concentration of 1.2 pmol and incubated with cells for 16 h in a 37 °C humidified chamber. If repeat FISH was performed, a CAG_10_ Alexa Fluor 594 probe (Stellaris) was included in hybridization buffer at a concentration of 380 ng/uL. After primary incubation, cells were washed with probe wash buffer (Molecular Instruments) 4X in a 37 °C humidified chamber, then twice with 5X SSC and 0.1% Tween-20 (SSCT), and finally pre-amplification was performed for 30 min at RT with amplification buffer (Molecular Instruments). During pre-amplification, appropriate HCR amplifier sets corresponding to primary probe initiators were heated separately in PCR tubes for 90 s at 95 °C and allowed to cool to RT in the dark. Primed amplifiers were then added to amplification buffer at a concentration of 2.4 pmol and incubated with cells for 4 h at RT. Cells were then washed 5X with 5X SSCT with DAPI (1 ng/uL) included on the final wash, mounted, and imaged.

### Fixed cell imaging

Results of HCR FISH in C2C12 myoblasts and N2A cells were imaged on a Zeiss LSM 880 Axio Observer microscope with Airyscan using a Plan-Apochromat 1.46 NA x100 oil objective and processed using Zeiss ZEN Black software (Version 14.0.15.201). Fixed cell imaging for centrosome recruitment assay was done with widefield illumination using a Plan-Apochromat 1.3 NA ×40 oil objective. Fixed cell imaging of the split kinesin assay in N2As and MBNL in primary neurons was performed using widefield illumination on a Nikon Eclipse TE300 inverted microscope with a Plan-Neofluar 1.4 NA x60 oil objective. For quantitative colocalization experiments, 12 images were taken in a z-series at 2 um steps and deconvolved using a 3-D blind constrained iterative algorithm (AutoQuant, CyberMetrics). Imaris imaging software (Bitplane), namely the ‘Coloc’ module, was then used for analysis on the deconvolved images. Quantitative colocalization analysis involved creating a 3-D mask of the MAP2 channel from select neurites and excluding background signal from outside this volume. Within the MAP2 masked volume, two measures of colocalization, Pearson’s Correlation Coefficient and Mander’s Overlap Coefficient, were calculated between HA-MBNL1 and the respective endogenous KIF signals.

### Live cell imaging

All imaging in C2C12 myoblasts was performed on a Zeiss LSM 880 Axio Observer microscope with Airyscan using a Plan-Apochromat 1.46 NA ×100 oil objective. To obtain sufficiently high signal to noise ratios and resolve individual RNPs, we imaged cells in the low levels of ponasterone A and no doxycycline, relying on low and leaky expression from inducible promoters for MS2 and MCP components. Myoblasts were cultured on chambered coverglass (Thermo Fisher, 155409) with 2 uM Ponasterone A overnight and incubated with JF646 HaloTag ligand (Promega, GA1120) 15 min before imaging. Fluorobrite media (Thermo Fisher, A1896701) containing no phenol red supplemented with 100 U/mL penicillin, 100 mg/mL streptomycin, and 10% fetal bovine serum was used to wash and culture unbound HaloTag ligand and minimize autofluorescence. Cells were then placed in a humidified chamber with 5% CO_2_ at 37 °C. Images were collected at 10 frames per second for 60 s. For imaging purposes, MCP-Halo expression was not induced by doxycycline due to noticeable, low-level expression observed in stable cells.

For live cell imaging of primary neurons, coverslips were transferred to a pre-warmed Chamlide CMB magnetic chamber (Quorum Technologies, CM-B18-1) and placed in warmed Hibernate E Low Fluorescence (BrainBits, HELF) CO_2_ independent media, supplemented with B27 Plus and GlutaMAX. Neurons were then supplemented with 100 ng/mL BDNF, a well-established paradigm used to induce transport of RNPs^96,108^, and imaged after at least 10 min with using widefield illumination on a Nikon Eclipse TE300 inverted microscope with a Plan-Neofluar 1.4 NA ×60 oil objective supplied with live cell imaging heating chamber. For imaging of motile EGFP-MBNL isoforms, a 200 ms exposure time was used with images acquired every 260 ms for 1–2 min. Granules were manually tracked in ImageJ and velocities were calculated by dividing total time by total distance of each track. For imaging of co-transport with EGFP-MBNL and kinesins, two color channel images were acquired every 1.15 sec for 1 min. Regions in either dendrites or axons were chosen where long processive movements (≥2 µm) were observed, then regions were converted to kymographs with ImageJ Reslice tool. Tracks were then labeled with a line ROI tool and measured. Distance and velocity were calculated on the basis of the line length and angle. Tracks that overlapped in both channels were categorized as co-transported particles.

### Centrosome recruitment analysis

N2A cells were transfected as described above with BicD2-KIF tail fusions and MBNL-EGFP proteins at a ratio of 2:3 in 4-well chamber slides (Thermo Fisher, 154526). 24 h after transfection, cells were fixed and processed for immunofluorescence as described above with a rabbit anti-FLAG antibody (1:1000, Cell Signaling Technologies, D6W5B) to visualize BicD2-KIF fusions. A collection of tile scans was taken from each condition on a Zeiss LSM 880 Axio Observer microscope with widefield illumination using a Plan-Apochromat 1.3 NA ×40 oil objective. After opening each tile scan image on ImageJ, cells and centrosomes were manually chosen, traced, and cataloged with the ROI Manager using MBNL-EGFP and centrosomal FLAG signal as a mask, respectively. Care was taken to analyze cells that were not overexpressing tail proteins and did not have abnormally asymmetric centrosomes. Centrosome recruitment was quantified by taking the log_2_ value of the average intensity of MBNL signal at the centrosome divided by the average MBNL intensity in the whole cell.

### RNA granule dynamics

Time lapse images were captured using a Plan-Apochromat 1.46 NA ×100 oil objective at 10 frames/second. Background fluorescence was corrected and spot signal enhanced through setting a rolling ball radius of 3 pixels. Particle tracks were analyzed with the Fiji (Version 7.7.2) TrackMate software^55^. RNP spots were identified based on approximate diameter (~0.5 µm), consistent with previous observations of RNP granule size^109,110^, and quality across each time series. Spot track length threshold was set at >20 frames (2 s). Spot location data was analyzed using custom scripts written in Python 3. Mean squared displacement (MSD) of each particle along the track was calculated at increasing time lags (up to lag time 7) and was used to quantify diffusion coefficients, which represents the slope of MSD plotted over lag time. Distance refers to the furthest point away from the track start a spot appears along the track.

### Neurite fractionation and RNA-seq

Neurite fractionation was performed similar to a previously established protocol^111^. The underside of hanging cell culture inserts with 1 µm pores (Corning, 353102) were coated with diluted Matrigel preparation (Corning, 354277) and placed into specialized, deep 6-well plates (Corning, 353502). 2 mL of 10% DMEM media was placed on top and underneath the hanging insert membrane prior to cell plating. N2A cells were then densely plated onto membranes at a concentration of 1 × 10^6^. After 24 h, serum-containing media was replaced with no-serum media and neurites were allowed to grow for 48 h. Subsequently, no-serum media was removed from both sides of the membrane, which was washed once with PBS, then cell bodies were collected with a cell scraper in 1 mL PBS. Membranes were separated from their housing and placed into a lysis buffer (Zymo, R1060). Cell body and neurite samples from all wells of a 6-well plate were combined separately for each condition. After collecting all samples, cell bodies were spun down at 2000 × *g* for 5 min and resuspended in 600 uL of PBS, with 100 uL reserved for RNA isolation. Reserved cell body volume was lysed, then cell body and neurite samples were processed for RNA isolation with a Quick-RNA Microprep Kit (Zymo, R1050). Following isolation, ribosomal RNA was depleted from 300 ng of the total RNA samples using the NEBNext rRNA Depletion Kit (NEB, E6310L), then processed for RNA-seq library prep with the NEBNext Ultra II Directional RNA Library Prep Kit (NEB, E7530L), and finally sequenced on an Illumina NextSeq 500. Fastq files were obtained using bcl2fastq2 v2.20. Gene expression values were quantitated by kallisto 0.43.0 (mapping to mm10 Refseq annotations) and normalized using the R “cyclicLoess” function. Localization ratios were computed by log_2_(neurite/soma). RNA-Seq data have been submitted to GEO (GSE207597).

### Membrane fractionation

Extraction of cellular fractions was performed using lysis buffer (10 mM piperazine-N,N′-bis(2-ethanesulfonic acid) (PIPES), 0.25 M sucrose, 1 mM triethylene glycol diamine tetraacetic acid (EGTA), 5 mM magnesium chloride (MgCl_2_), and 25 mM NaCl) supplemented with 0.015% digitonin (cytosolic fraction) or 1% Triton X-100 (membrane fraction). Fractions were collected by incubating cells in 6-well plates with 500 uL cytosolic lysis buffer for 5 min at RT, collecting cytosolic sample, then incubating with membrane lysis buffer for 5 min at RT followed by collection of membrane fraction. Protein samples were processed by diluting in 4X LDS sample buffer (mentioned previously). RNA was isolated from the same samples using Direct-Zol RNA Miniprep Kit (Zymo, R2050) and iScript cDNA synthesis kit used to make cDNA (Bio-Rad, 1708890). Real-time amplification of the SunTag coding sequence, Gapdh (cytosolic), and Calnexin (membrane) using qPCR primers (IDT) with Q5 polymerase (New England Biolabs, M0492) was measured with a highly sensitive dsDNA detection dye (Lumiprobe, 11010) on a 96-well RT-PCR thermal cycler (Bio-Rad).

### Reporting summary

Further information on research design is available in the Nature Portfolio Reporting Summary linked to this article.

## Supplementary information

Supplementary Information

Description of Additional Supplementary Files

Supplementary Data 1

Supplementary Data 2

Supplementary Data 3

Supplementary Data 4

Supplementary Movie 1

Supplementary Movie 2

Supplementary Movie 3

Supplementary Movie 4

Supplementary Movie 5

Reporting Summary

## Data Availability

The data that support this study are available from the corresponding authors upon reasonable request. Data pertaining to RNA sequencing experiments and GTEx kinesin expression have been included in this paper. Source data for neurite fractionation experiments can be found on Gene Expression Omnibus (accession GSE207597). Source data are provided with this paper.

## Source data

Source Data

## Code availability

Custom Python 3 script to quantify particle tracking data from TrackMate files can be found on Zenodo (10.5281/zenodo.7865793).
