# Supplementary material for: Muscleblind-like proteins use modular domains to localize RNAs by riding kinesins and docking to membranes
Source: Nat Commun. 2023 Jun 9;14:3427. doi: 10.1038/s41467-023-38923-6 (PMC10256740; doi:10.1038/s41467-023-38923-6)
Supplement: Supplementary file 1 — Supplementary Information [file 41467_2023_38923_MOESM1_ESM.pdf]

Supplementary Information

Ryan P. Hildebrandt[1*], Kathryn R. Moss[2*], Aleksandra Janusz-Kaminska[2], Luke A. Knudson[2], Lance T. Denes[1], Tanvi Saxena[1], Devi Prasad Boggupalli[1], Zhuangyue Li[1], Kun Lin[2], Gary J. Bassell[2‡], Eric T. Wang[1,3‡]

[1]Department of Molecular Genetics & Microbiology, Center for Neurogenetics, Genetics Institute, University of Florida, Gainesville, FL
[2]Department of Cell Biology, Emory University School of Medicine, Atlanta, GA
[3]Myology Institute, University of Florida, Gainesville, FL

These authors contributed equally*: Ryan P. Hildebrandt, Kathryn R. Moss
Co-corresponding authors‡: Gary J. Bassell (gary.bassell@emory.edu), Eric T. Wang (eric.t.wang@ufl.edu)

Supplementary Figure 1

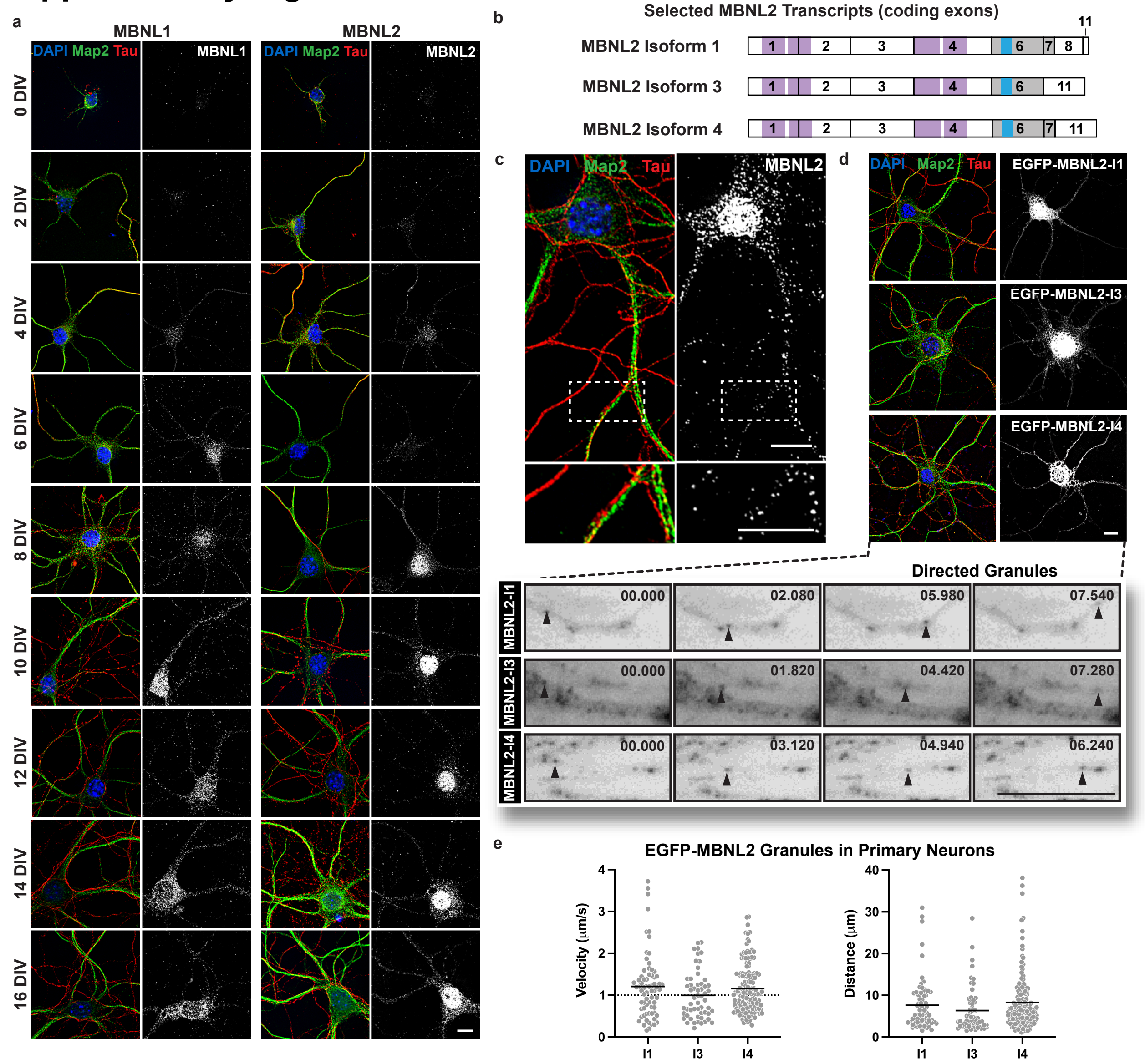

MBNL1 and -2 are developmentally regulated in primary cortical neurons and cytoplasmic MBNL2 isoforms are actively transported in axons and dendrites.
a, Mouse primary cortical neurons were cultured for 0-16 DIV and fixed every other day. Immunofluorescence of MBNL1 and MBNL2 granules (white) in axons (Tau, red) and dendrites (Map2, green) corresponding to DIV. **b**, Exon and protein domain structure of cytoplasmic MBNL2 isoforms studied. nuclei (blue) labeled with DAPI. Scale bar = 10 µm. **c**, Representative image showing MBNL2 granules (white) in axons (Tau, red), dendrites (Map2, green), and nuclei (blue) of 9 DIV cultured primary mouse cortical neurons. **d**, Representative images of EGFP-MBNL2-I1, -I2, and -I3 isoforms (white) in 9 DIV cultured primary mouse cortical neurons. Axons and dendrites labeled with Tau (red) and Map2 (green), respectively. Below are representative time lapse images of motile EGFP-MBNL2 isoform granules in live mouse cortical neurons. Time (sec) indicated in top right. **e**, Quantitation of speeds and distances traveled by cytoplasmic MBNL2 granules (MBNL2-I1 - 70 tracks, 2.8 tracks/cell; MBNL2-I3 - 56 tracks, 1.87 tracks/cell; MBNL2-I4 - 141 tracks, 3.62 tracks/cell). Tracks shown come from 3 biological replicates. Dotted line in velocity chart represents typical speed of microtubule-dependent transport. Bars represent mean. Nuclei labeled with DAPI (blue). Scale bars = 10 µm.

Supplementary Figure 2

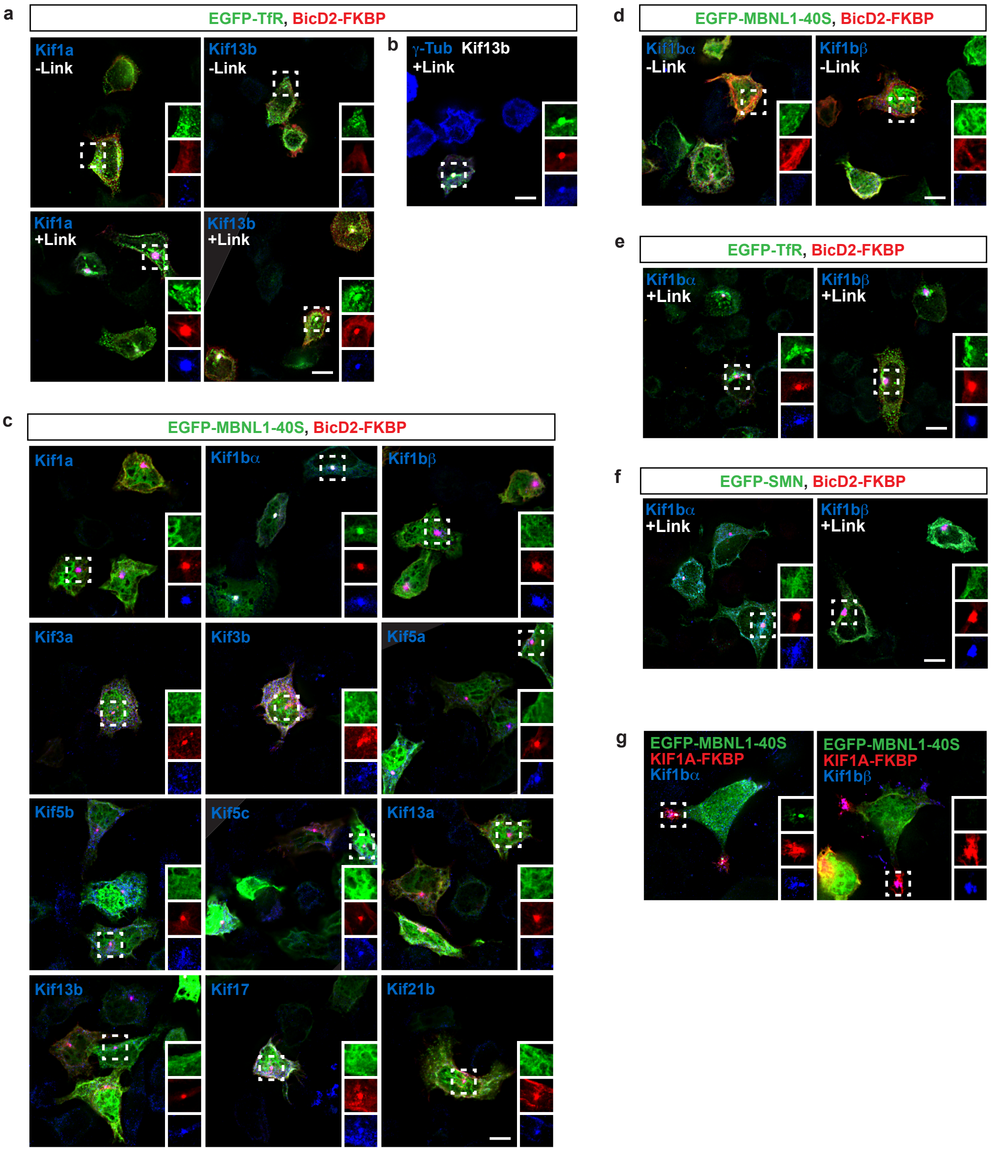

Kinesin tail screen with MBNL1 and controls.
a, N2A cells were transfected with EGFP-TfR (green), tdTomato-tagged BicD2-FKBP (red) and Myc-Kif1a-FRB or Myc-Kif13b-FRB in the presence of linker drug or 100% ethanol after ~16 hours. Cells were fixed after 3 hours and processed for immunofluorescence with an A) Myc or **b**, γ-Tubulin antibody (blue). **c**, N2A cells were transfected with EGFP-tagged MBNL1-40S (green), tdTomato-tagged BicD2-FKBP (red) and Myc-tagged kinesin tails (Kif1a-FRB, Kif1b α-FRB, Kif1bβ-FRB, Kif3a-FRB, Kif3b-FRB, Kif5a-FRB, Kif5b-FRB, Kif5c-FRB, Kif13a-FRB, Kif13b-FRB, Kif17-FRB or Kif21b-FRB in the presence of linker drug. Cells were fixed after 3 hours and processed for immunofluorescence with a Myc antibody (blue). **d**, N2A cells were transfected with EGFP-tagged MBNL1-40S (green), tdTomato-tagged BicD2-FKBP (red) and Myc-Kif1bα-FRB or Myc-Kif1bβ-FRB in the presence of 100% ethanol or **e**, linker drug. **f**, EGFP-tagged SMN (green) was transfected with tdTomato-tagged BicD2-FKBP (red) and Myc-Kif1bα-FRB or Myc-Kif1bβ-FRB in the presence of linker drug. **g**, N2A cells were transfected with EGFP-tagged MBNL1-40S (green), tdTomato-tagged KIF1A-FKBP (red) and Myc-Kif1b α-FRB or Myc-Kif1bβ-FRB in the presence of linker drug. Scale bars = 10 μm.

Supplementary Figure 3

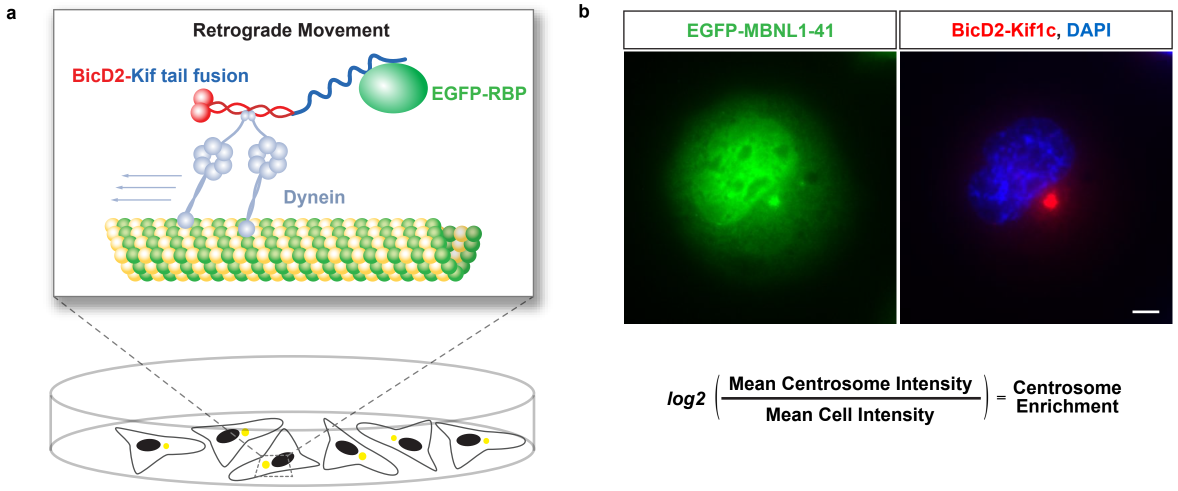

Schematic of the centrosome recruitment assay and quantitation.
a, Schematic of the assay components and dynein engagement towards the centrosome. **b**, Representative images of a EGFP-MBNL1-41 (green) when co-expressed with a FLAG-BicD2-Kif1c tail fusion (red). Nuclei (blue) labeled with DAPI. Scale bar = 5 μm. Centrosome enrichment is defined as the $\log_2$ value of the mean centrosome intensity divided by the mean cell intensity of the transported cargo.

Supplementary Figure 4

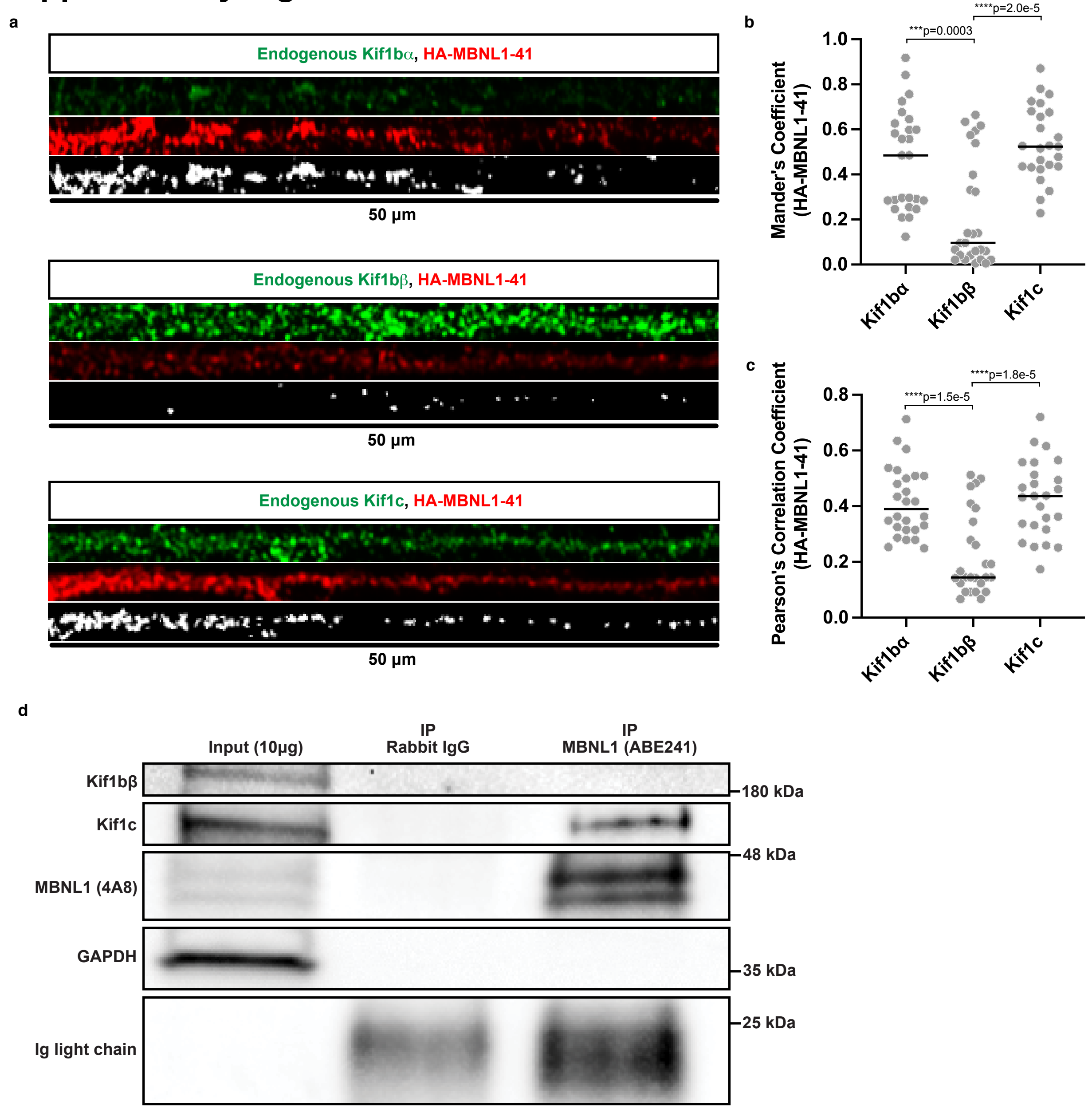

Endogenous Kif1bα and Kif1c co-localize with HA-MBNL1-41.
a, Representative 50 µm neurite scans with immunofluorescence for endogenous kinesins (green) and transduced HA-MBNL1-41 (red) in 12 DIV primary mouse cortical neurons. Colocalized signal is represented below each condition (white). **b**, Mander's overlap coefficient of endogenous kinesins with HA-MBNL1 and **c**, Pearson's correlation coefficient of endogenous kinesins with HA-MBNL1-41. Bars denote median. Kif1bα: n=26 neurites, Kif1bβ: n=26 neurites, Kif1c: n=25 neurites. Experiments performed across 3 biological replicates. **d**, Representative western blots against endogenous Kif1bβ, Kif1c, MBNL1, and GAPDH following immunoprecipitation of endogenous MBNL1. ***p<0.001, ****p<0.0001, Two-tailed Mann-Whitney U test. Source data provided as source data file.

Supplementary Figure 5

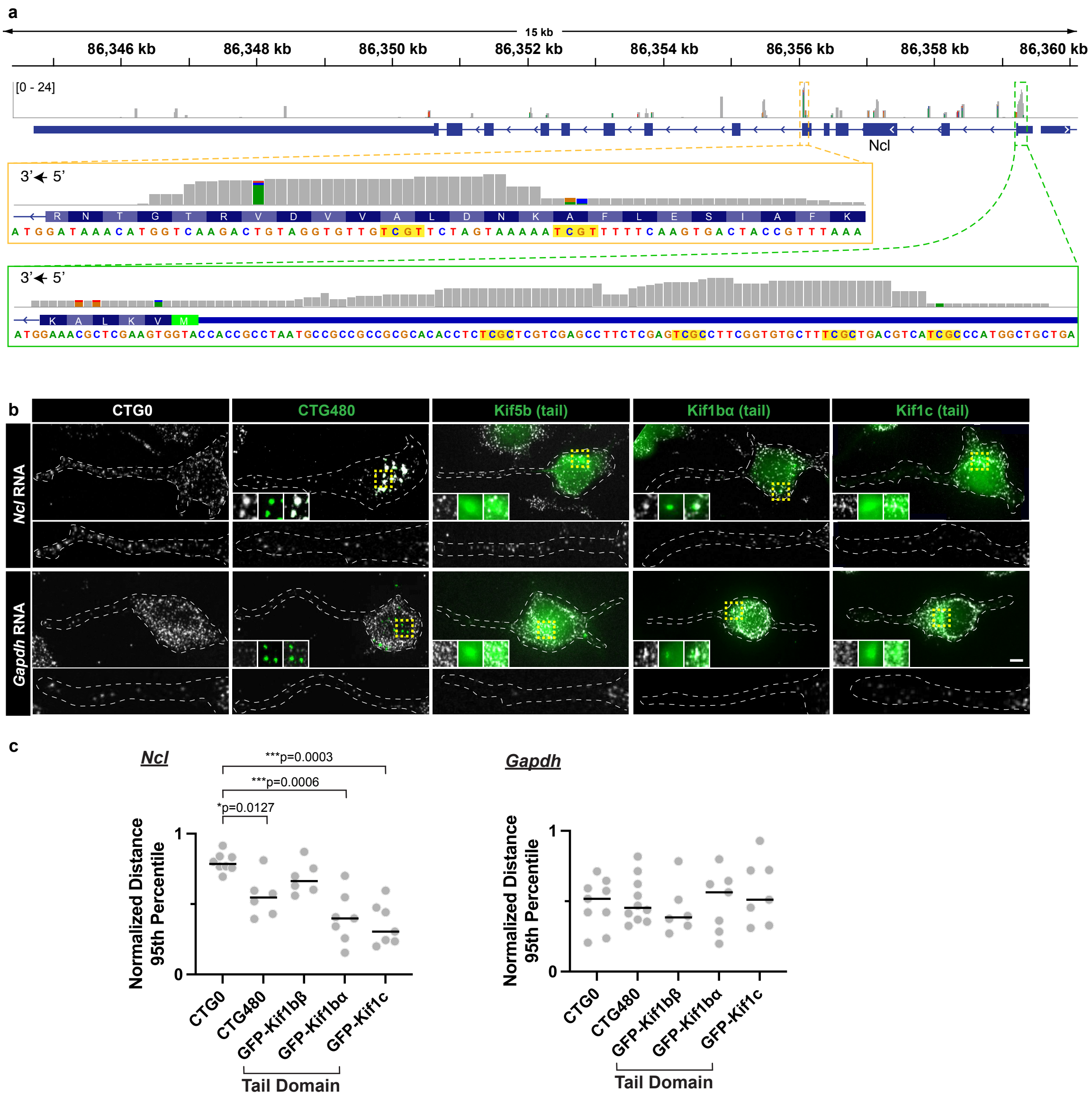

HCR FISH in differentiated CAD cells confirms Ncl mislocalization.
a, MBNL1 CLIP from C2C12 myoblasts at the Ncl locus (mm10). Insets show two specific locations within the coding sequence (orange box) and 5' UTR (green box), in which YGCY motifs are highlighted in yellow. **b**, Representative HCR FISH images for Ncl and Gapdh (red) with CTG repeats or dominant negative kinesin tails (green). Scale bar = 10 µm. **c**, Distances of Ncl and Gapdh HCR FISH spots from the soma of each cell across conditions. Distances were normalized as a fraction of total neurite length, and the 95th percentile of distances within each cell is plotted as an individual gray circle. Each cell (Ncl - CTG: n=1 cells, CTG48: n=1 cells, Kif5b: n=7 cells, Kif1bα: n=7 cells, Kif1c: n=7 cells; Gapdh - CTG0: n=10 cells, CTG480: n=10 cells, Kif15b: n=6 cells, Kif1bαn=7 cells, Kif1c: n=7 cells; across 3 biological replicates) contained >200 Ncl spots and >150 Gapdh spots. Bars represent the median across cells. *p<0.05, ***p<0.001, Two-tailed Mann-Whitney U test. Source data provided as source data file.
