# Supplementary material for: Muscleblind-like proteins use modular domains to localize RNAs by riding kinesins and docking to membranes
Source: Nat Commun. 2023 Jun 9;14:3427. doi: 10.1038/s41467-023-38923-6 (PMC10256740; doi:10.1038/s41467-023-38923-6)
Supplement: Supplementary file 2 — Description of Additional Supplementary Files [file 41467_2023_38923_MOESM2_ESM.pdf]

Description of Additional Supplementary Files

File Name: Supplementary Data 1
Description: TPM values from sequencing of neurite fractionation samples.

File Name: Supplementary Data 2
Description: Filtered log2 LR values of all conditions along with number of annotated MBNL1 CLIP sites in mouse brain.

File Name: Supplementary Data 3
Description: Kinesin transcript expression across tissues (average values for all samples in each tissue) as assessed by RNA-seq from GTEX.

File Name: Supplementary Data 4
Description: Full plasmid sequences used in this study.

File Name: Supplementary Movie 1
Description: **EGFP-MBNL1 granules in live cultured neurons.** Cytoplasmic, EGFP-tagged MBNL1-40 and -41 kDa isoforms were transfected by magnetofection into live cortical mouse neurons. Granules (black) exhibit directed motion. Particle track overlays seen in blue. 24 frames per second. Scale bars = 5 μm.

File Name: Supplementary Movie 2
Description: **EGFP-MBNL1 granules in live C2C12 myoblasts.** Cytoplasmic, EGFP-tagged MBNL1-40 kDa and -41 kDa isoform granules (white) stably expressed in live mouse C2C12 myoblasts. Nuclear, EGFP-tagged MBNL1-42 kDa and -43 kDa isoforms are observed are confined to the nucleus. Cytoplasmic granules exhibit mostly anchored behavior, with occasional diffusive or directed motion. 60 frames per second. Scale bars = 5 μm.

File Name: Supplementary Movie 3
Description: **Representative MCP-Halo particle track output from TrackMate.** Cropped particle track output in TrackMate Fiji plugin from MCP-Halo RNP stably expressed in C2C12 myoblasts. 60 frames per second. Scale bar = 1 μm.

File Name: Supplementary Movie 4
Description: **Representative particle tracks of MCP-Halo granules.** Representative movies of C2C12 myoblasts expressing MCP-Halo-MBNL1-RIM, -ΔC RIM, and -Δ3, ΔC RIM (left) with track overlays from TrackMate Fiji plugin (right). Particle tracks from full-length RIM exhibit a greater confinement and anchoring compared to ΔC RIM and Δ3, ΔC RIM that lack the MBNL1 C-terminal tail domain. 60 frames per second. Scale bars = 5 μm.

File Name: Supplementary Movie 5
Description: **MCP-Halo protein and MS2 reporter are necessary to form visible granules.** Representative movies of C2C12 myoblasts expressing MCP-Halo alone or with the C-terminal tail domain (+C-terminus) and with (+MS2) or without (no MS2) a 45xMS2 reporter. Presence

of the reporter is required to form visible RNP granules. 60 frames per second. Scale bars = 5 μm.
