# Supplementary material for: Muscleblind-like proteins use modular domains to localize RNAs by riding kinesins and docking to membranes
Source: Nat Commun. 2023 Jun 9;14:3427. doi: 10.1038/s41467-023-38923-6 (PMC10256740; doi:10.1038/s41467-023-38923-6)
Supplement: Supplementary file 12 — Reporting Summary [file 41467_2023_38923_MOESM12_ESM.pdf]

nature portfolio

Corresponding author(s): Eric T. Wang

Last updated by author(s): Apr 17, 2022

Reporting Summary

Nature Portfolio wishes to improve the reproducibility of the work that we publish. This form provides structure for consistency and transparency in reporting. For further information on Nature Portfolio policies, see our Editorial Policies and the Editorial Policy Checklist.

Statistics

For all statistical analyses, confirm that the following items are present in the figure legend, table legend, main text, or Methods section.

n/a	Confirmed	
☐	☒	The exact sample size (*n*) for each experimental group/condition, given as a discrete number and unit of measurement
☐	☒	A statement on whether measurements were taken from distinct samples or whether the same sample was measured repeatedly
☐	☒	The statistical test(s) used AND whether they are one- or two-sided<br>*Only common tests should be described solely by name; describe more complex techniques in the Methods section.*
☒	☐	A description of all covariates tested
☐	☒	A description of any assumptions or corrections, such as tests of normality and adjustment for multiple comparisons
☐	☒	A full description of the statistical parameters including central tendency (e.g. means) or other basic estimates (e.g. regression coefficient) AND variation (e.g. standard deviation) or associated estimates of uncertainty (e.g. confidence intervals)
☐	☒	For null hypothesis testing, the test statistic (e.g. *F*, *t*, *r*) with confidence intervals, effect sizes, degrees of freedom and *P* value noted<br>*Give P values as exact values whenever suitable.*
☒	☐	For Bayesian analysis, information on the choice of priors and Markov chain Monte Carlo settings
☒	☐	For hierarchical and complex designs, identification of the appropriate level for tests and full reporting of outcomes
☐	☒	Estimates of effect sizes (e.g. Cohen's *d*, Pearson's *r*), indicating how they were calculated

Our web collection on statistics for biologists contains articles on many of the points above.

Software and code

Policy information about availability of computer code

Data collection	Live cell particle tracking images were collected using the Zeiss Zen Black Software (Version 14.0.15.201). Individual particle tracks were collected using Fiji/ImageJ TrackMate software (Version 7.7.2).
Data analysis	All images were processed using ImageJ (Version 2.3.0 /1. 53 q). Particle tracking analysis was performed using a custom Python 3 script (available upon request). All graphs were generated using Prism (Version 9.3.1).

For manuscripts utilizing custom algorithms or software that are central to the research but not yet described in published literature, software must be made available to editors and reviewers. We strongly encourage code deposition in a community repository (e.g. GitHub). See the Nature Portfolio guidelines for submitting code & software for further information.

Data

Policy information about availability of data

All manuscripts must include a data availability statement. This statement should provide the following information, where applicable:

- Accession codes, unique identifiers, or web links for publicly available datasets
- A description of any restrictions on data availability
- For clinical datasets or third party data, please ensure that the statement adheres to our policy

Data pertaining to RNA sequencing experiments and GTEx kinesin expression have been included in this paper. Source data for neurite fractionation experiments can be found on Gene Expression Omnibus (accession GSE207597).

Human research participants

Policy information about studies involving human research participants and Sex and Gender in Research.

Reporting on sex and gender	N/A
Population characteristics	N/A
Recruitment	N/A
Ethics oversight	N/A

Note that full information on the approval of the study protocol must also be provided in the manuscript.

Field-specific reporting

Please select the one below that is the best fit for your research. If you are not sure, read the appropriate sections before making your selection.

☒ Life sciences ☐ Behavioural & social sciences ☐ Ecological, evolutionary & environmental sciences

For a reference copy of the document with all sections, see nature.com/documents/nr-reporting-summary-flat.pdf

Life sciences study design

All studies must disclose on these points even when the disclosure is negative.

Sample size	No statistical methods were used to determine sample size. For live cell imaging and centrosome recruitment experiments, all conditions were repeated at least 3 times with care taken to maximize observations of transported cargoes and obtain accurate measurements of data distribution. For immunoprecipitation, western blot, fluorescence in situ hybridization, and qPCR, all experiments were repeated at least 3 times to obtain accurate measurements on significant differences between conditions. Neurite fractionation experiment was performed once because it was intended to be used as a tool for determining transcripts that deserved scrutiny with FISH methods; changes in LR values were analyzed by correlating to MBNL CLIP tag density and using statistical methods for small sample sizes (Fisher's Exact Test).
Data exclusions	No data were excluded.
Replication	All experiments were replicated at least 3 times, with the exception of neurite fractionation experiments. Neurite fractionation was performed once as a tool for downstream confirmation of mis-localized or non-affected transcripts, which was confirmed through FISH (Ncl and Gapdh). All replications were successful.
Randomization	For all relevant experiments, samples were randomly allocated to each condition. Cells used for imaging were selected based simply on expression or differentiation status and image collection was performed by experimenters in an unbiased manner through tile scan (centrosome recruitment) and time series collection (particle tracking/kinesin co-transport).
Blinding	Blinding was not applicable, as investigators were aware of conditions during analysis, however, data collection was performed in an unbiased method. During live cell imaging collection, it is unknown how particles will behave throughout a time series. For centrosome recruitment analysis, tile scan collection ensured investigators are unaware of the cell state.

Reporting for specific materials, systems and methods

We require information from authors about some types of materials, experimental systems and methods used in many studies. Here, indicate whether each material, system or method listed is relevant to your study. If you are not sure if a list item applies to your research, read the appropriate section before selecting a response.

Materials & experimental systems

n/a	Involved in the study	
☐	☒	Antibodies
☐	☒	Eukaryotic cell lines
☒	☐	Palaeontology and archaeology
☐	☒	Animals and other organisms
☒	☐	Clinical data
☒	☐	Dual use research of concern

Methods

n/a	Involved in the study	
☒	☐	ChIP-seq
☒	☐	Flow cytometry
☒	☐	MRI-based neuroimaging

Antibodies

Antibodies used

monoclonal mouse anti-MBNL1 (MB1a(4A8)), Developmental Studies Hybridoma Bank, dilution 1:100)
monoclonal mouse anti-MBNL2 (MB2a(3B4)), Developmental Studies Hybridoma Bank, dilution 1:100)
polyclonal rabbit anti-Map2 (AB5622, Millipore-Sigma, dilution 1:1000)
polyclonal guinea pig anti-Map2 (188 004, Synaptic Systems, dilution 1:1000)
polyclonal chicken anti-Tau (TAU, Aves Labs, dilution 1:1000)
monoclonal mouse anti-Myc (9E10, Developmental Studies Hybridoma Bank, dilution 1:100)
monoclonal mouse anti-γ-Tubulin (ab11316, Abcam, dilution 1:500)
monoclonal rabbit anti-FLAG (14793S, Cell Signaling Technologies, dilution 1:1000)
monoclonal mouse anti-KIF1B - recognizes KIF1Bβ (clone 10F3.1, MABC309, EMD Millipore, dilution 1:50)
monoclonal mouse anti-KIF1B - recognizes KIF1Bα (sc-376246, Santa Cruz, dilution 1:50)
polyclonal rabbit anti-KIF1C (ab125903, Abcam, dilution 1:200)
monoclonal rabbit anti-HA (3724T, Cell Signaling Technologies, dilution 1:800)
monoclonal mouse anti-HA (clone 16B12, 901501, BioLegend, dilution 1:1000)
monoclonal mouse anti-Hsp90 (ab13492, Abcam, dilution 1:1000)
polyclonal rabbit anti-Calnexin (ab22595, Abcam, dilution 1:1000)
polyclonal chicken anti-GFP (ab13970, Abcam, dilution 1:5000)
polyclonal rabbit anti-β-Actin (4967, Cell Signaling Technologies, dilution 1:2000)
polyclonal rabbit anti-MBNL1 antibody (ABE241, Millipore Sigma, dilution 1:1000)
monoclonal mouse anti-RFP antibody (5F8, Proteintech, dilution 1:1000)
polyclonal rabbit anti-normal IgG (12-370, Millipore Sigma, 3 ug/200 uL)
Rhodamine-conjugated anti-GAPDH human Fab fragment (12004168, Bio-Rad, dilution 1:1000)
goat anti-mouse Alexa Fluor 488 (A11001, Thermo Fisher, dilution 1:500)
goat anti-mouse Alexa Fluor 647 (A21235, Thermo Fisher, dilution 1:500)
goat anti-rabbit Alexa Fluor 647 (A32733, Thermo Fisher, dilution 1:1000)
goat anti-chicken Alexa Fluor 633 (A21103, Thermo Fisher, dilution 1:500)
goat anti-chicken Alexa Fluor 633 (A21105, Thermo Fisher, dilution 1:500)
IRDye 680LT donkey anti-rabbit, (#926-68073, LICOR, dilution 1:5000)
IIRDye 680LT donkey anti-mouse, (#926-68072, LICRO, dilution 1:5000)
IRDye 800CW donkey anti-rabbit, (#926-32213, LICOR, dilution 1:5000)
IRDye 800CW donkey anti-chicken (#926-32218, LICOR, dilution 1:5000)

Validation

anti-MBNL1 (MB1a(4A8), made in SP2/0 cells, epitope corresponds to VSPSL /linker sequence in MBNL1 exon 3) initially confirmed in Holt et al (2007), PMID: 17825047
anti-MBNL2 (MB2a(3B4), made in SP2/0 cells, epitope PYLA) initially confirmed in Holt et al (2009), PMID: 19095965
rabbit anti-Map2 (AB5622) tested for quality assurance by Millipore-Sigma through immunohistochemistry and has been cited by 55 publications on https://www.emdmillipore.com/US/en/product/Anti-Microtubule-Associated-Protein-2-MAP2-Antibody,MM_NF-AB5622#anchor_BRO
guinea pig anti-Map2 (188 004) tested by immunocytochemistry and has 113 citations on https://sysy.com/product/188004
chicken anti-Tau (TAU) antibody mixture tested for specificity by immunohistochemistry
monoclonal mouse anti-Myc (9E10, derived from SP2 cells, epitope EQKLISEEDL) initially confirmed by Evan et al (1985), PMID: 3915782 and has been cited by 51 publications on https://dshb.biology.uiowa.edu/9E-10
monoclonal mouse anti-γ-Tubulin (ab11316, clone GTU-88) was confirmed by Abcam through immunocytochemistry and has been cited by 150 publications on https://www.abcam.com/gamma-tubulin-antibody-gtu-88-centrosome-marker-ab11316.html
monoclonal rabbit anti-FLAG (clone D6W5B) has been verified by immunofluorescence and has been cited by 701 publications on (https://www.cellsignal.com/products/primary-antibodies/dykddddk-tag-d6w5b-rabbit-mab-binds-to-same-epitope-as-sigma-s-anti-flag-m2-antibody/14793
monoclonal mouse anti-KIF1B - recognizes KIF1Bβ (MABC309, clone 10F3.1) tested for isoform specificity by western blot by Millipore-Sigma (https://www.emdmillipore.com/US/en/product/Anti-KIF1B-Antibody-clone-10F3.1,MM_NF-MABC309) and in this manuscript
monoclonal mouse anti-KIF1B - recognizes KIF1Bα (sc-376246, clone E-12) tested by western blot in this manuscript, antigen corresponds to unique C-terminal tail sequence of the alpha isoform https://www.scbt.com/p/kif1b-antibody-e-12
polyclonal rabbit anti-KIF1C (ab125903) was confirmed by Abcam through western blot and validated for use by Denes et al., 2022, PMID: 34707124
monoclonal rabbit anti-HA (3724T, clone C29F4) has been confirmed through immunofluorescence and cited by 1983 publications on https://www.cellsignal.com/products/primary-antibodies/ha-tag-c29f4-rabbit-mab/3724
monoclonal mouse anti-HA (clone 16B12, 901501) has been tested for various applications by 83 publications https://www.biolegend.com/fr-ch/products/purified-anti-ha-11-epitope-tag-antibody-11374
monoclonal mouse anti-Hsp90 (ab13492) has been verified by Abcam for western blot and has been cited by 89 publications on https://www.abcam.com/hsp90-antibody-ac88-ab13492.html
polyclonal rabbit anti-Calnexin (ab22595) has been verified by Abcam for western blot and has been cited by 291 publications on https://www.abcam.com/calnexin-antibody-er-marker-ab22595.html
polyclonal chicken anti-GFP (ab13970) has been verified by Abcam for western blot and been cited by 2684 publications on https://www.abcam.com/gfp-antibody-ab13970.html
polyclonal rabbit anti-β-Actin (4967) has been verified by Cell Signaling for western blot and cited by 2758 publications on https://www.cellsignal.com/products/primary-antibodies/b-actin-antibody/4967
polyclonal rabbit anti-MBNL1 antibody (ABE241) was evaluated for specificity by Millipore-Sigma through western blot
monoclonal mouse anti-RFP antibody (5F8) has been tested through immunofluorescence by 239 publications on https://www.ptglab.com/products/RFP-antibody-5F8.htm
polyclonal rabbit anti-normal IgG antibody (12-370) is regularly evaluated for quality by Millipore Sigma. https://www.emdmillipore.com/US/en/product/Normal-Rabbit-IgG,MM_NF-12-370
Rhodamine-conjugated anti-GAPDH human Fab fragment (12004168, clone AbD22549) has been optimized for use in western blot as

a fluorescent primary antibody by Bio-Rad https://www.bio-rad.com/en-us/sku/12004168-hfab-rhodamine-anti-gapdh-primary-antibody-40-ul?ID=12004168
goat anti-mouse Alexa Fluor 488 (A11001, RRID AB_2534069) validated by Thermo Fisher with 870 citations https://www.thermofisher.com/antibody/product/Goat-anti-Mouse-IgG-H-L-Highly-Cross-Adsorbed-Secondary-Antibody-Polyclonal/A32723
goat anti-mouse Alexa Fluor 647 (A21235, RRID AB_2535804) validated by Thermo Fisher with 1308 citations on https://www.thermofisher.com/antibody/product/Goat-anti-Mouse-IgG-H-L-Cross-Adsorbed-Secondary-Antibody-Polyclonal/A-21235
goat anti-rabbit Alexa Fluor 647 (A32733, RRID AB_2633282) validated by Thermo Fisher with 403 citations https://www.thermofisher.com/antibody/product/Goat-anti-Rabbit-IgG-H-L-Highly-Cross-Adsorbed-Secondary-Antibody-Polyclonal/A32733
goat anti-chicken Alexa Fluor 633 (A21103, RRID AB_2535756) validated by Thermo Fisher with 85 citations https://www.thermofisher.com/antibody/product/Goat-anti-Chicken-IgY-H-L-Cross-Adsorbed-Secondary-Antibody-Polyclonal/A-21103
goat anti-chicken Alexa Fluor 633 (A21105, RRID AB_2535757) validated by Thermo Fisher with 78 citations https://www.thermofisher.com/antibody/product/Goat-anti-Guinea-Pig-IgG-H-L-Highly-Cross-Adsorbed-Secondary-Antibody-Polyclonal/A-21105
IRDye 680LT donkey anti-rabbit, (#926-68073, RRID AB_10954442) LICOR validates specificity of their antibodies through affinity chromatography and ELISA
IIRDye 680LT donkey anti-mouse, (#926-68072, RRID AB_10953628) LICOR validates specificity of their antibodies through affinity chromatography and ELISA
IRDye 800CW donkey anti-rabbit, (#926-32213, RRID AB_621848) LICOR validates specificity of their antibodies through affinity chromatography and ELISA
IRDye 800CW donkey anti-chicken (#926-32218, RRID AB_1850023) LICOR validates specificity of their antibodies through affinity chromatography and ELISA

Eukaryotic cell lines

Policy information about cell lines and Sex and Gender in Research

Cell line source(s)	C2C12 (ATCC, CCL-1772) cells were obtained from American type Culture Collection (ATCC) . Neuro2a (ATCC, CCL-131) cells and CAD (European Collection of Authenticated Cell Lines, 08100805) cells were obtained as a gift from the corresponding author's previous appointment in the lab of Dr. Chris Burge (Massachusetts Institute of Technology). Double knockout fibroblasts were a gift from Dr. Maury Swanson (University of Florida).
Authentication	Neuro2a cells and CAD cells were validated by differentiation achieved via serum starvation. C2C12 cells were validated for ability to fuse and form tubes. Fibroblasts were not validated.
Mycoplasma contamination	Neuro2a, C2C12, and mouse fibroblast cell lines tested negative for mycoplasma. CAD cells, used only for follow-up confirmation of mis-localization observed in Neuro2a cells (negative), tested positive (Extended Data Figure 5).
Commonly misidentified lines (See ICLAC register)	No misidentified lines were used in this work.

Animals and other research organisms

Policy information about studies involving animals; ARRIVE guidelines recommended for reporting animal research, and Sex and Gender in Research

Laboratory animals	Embryos were collected from pregnant C57BL6 mice at E17.
Wild animals	No wild animals were used in this study.
Reporting on sex	Sex was not determined, cells were isolated from embryos.
Field-collected samples	No field samples were collected.
Ethics oversight	The Emory University Institutional Animal Care and Use Committee (IACUC) provided oversight on the humane and ethical treatment of animals used in this study.

Note that full information on the approval of the study protocol must also be provided in the manuscript.
